# Transcriptomic Signatures of Progression to Tuberculosis Disease Among Close Contacts in Brazil

**DOI:** 10.1093/infdis/jiae237

**Published:** 2024-05-06

**Authors:** Simon C Mendelsohn, Bruno B Andrade, Stanley Kimbung Mbandi, Alice M S Andrade, Vanessa M Muwanga, Marina C Figueiredo, Mzwandile Erasmus, Valeria C Rolla, Prisca K Thami, Marcelo Cordeiro-Santos, Adam Penn-Nicholson, Afranio L Kritski, Mark Hatherill, Timothy R Sterling, Thomas J Scriba, the RePORT–South Africa and RePORT–Brazil Consortia, Nicole Bilek, Yolundi Cloete, Mzwandile Erasmus, Michelle Fisher, Katie Hadley, Rieyaat Hassiem, Mark Hatherill, Lungisa Jaxa, Stanley Kimbung Mbandi, Simon C Mendelsohn, Faheemah Meyer, Vanessa M Muwanga, Onke Nombida, Adam Penn-Nicholson, Rodney Raphela, Thomas J Scriba, Alison September, Timothy R Sterling, Prisca K Thami, Ashley Veldsman, Alice Andrade, Bruno B Andrade, Brenda Carvalho, Marcelo Cordeiro-Santos, Marina Cruvinel Figueiredo, Adriano Gomes, Afranio L Kritski, Valeria C Rolla, Timothy R Sterling

**Affiliations:** South African Tuberculosis Vaccine Initiative, Institute of Infectious Disease and Molecular Medicine, and Division of Immunology, Department of Pathology, University of Cape Town, Cape Town, South Africa; Laboratório de Pesquisa Clínica e Translacional, Instituto Gonçalo Moniz, Fundação Oswaldo Cruz, Salvador, Brazil; South African Tuberculosis Vaccine Initiative, Institute of Infectious Disease and Molecular Medicine, and Division of Immunology, Department of Pathology, University of Cape Town, Cape Town, South Africa; Laboratório de Pesquisa Clínica e Translacional, Instituto Gonçalo Moniz, Fundação Oswaldo Cruz, Salvador, Brazil; South African Tuberculosis Vaccine Initiative, Institute of Infectious Disease and Molecular Medicine, and Division of Immunology, Department of Pathology, University of Cape Town, Cape Town, South Africa; Vanderbilt University Medical Center, Nashville, Tennessee; South African Tuberculosis Vaccine Initiative, Institute of Infectious Disease and Molecular Medicine, and Division of Immunology, Department of Pathology, University of Cape Town, Cape Town, South Africa; Laboratorio de Pesquisa Clinica em Micobacterioses, Instituto Nacional de Infectologia Evandro Chagas, Fiocruz, Rio de Janeiro, Brazil; South African Tuberculosis Vaccine Initiative, Institute of Infectious Disease and Molecular Medicine, and Division of Immunology, Department of Pathology, University of Cape Town, Cape Town, South Africa; Fundação de Medicina Tropical Doutor Heitor Vieira Dourado, Manaus, Brazil; South African Tuberculosis Vaccine Initiative, Institute of Infectious Disease and Molecular Medicine, and Division of Immunology, Department of Pathology, University of Cape Town, Cape Town, South Africa; FIND, Geneva, Switzerland; Centro de Pesquisa em Tuberculose, Universidade Federal do Rio de Janeiro, Rio de Janeiro, Brazil; South African Tuberculosis Vaccine Initiative, Institute of Infectious Disease and Molecular Medicine, and Division of Immunology, Department of Pathology, University of Cape Town, Cape Town, South Africa; Vanderbilt University Medical Center, Nashville, Tennessee; South African Tuberculosis Vaccine Initiative, Institute of Infectious Disease and Molecular Medicine, and Division of Immunology, Department of Pathology, University of Cape Town, Cape Town, South Africa

**Keywords:** biomarkers, blood, prognostic, transcriptomic, tuberculosis

## Abstract

**Background:**

Approximately 5% of people infected with *Mycobacterium tuberculosis* progress to tuberculosis (TB) disease without preventive therapy. There is a need for a prognostic test to identify those at highest risk of incident TB so that therapy can be targeted. We evaluated host blood transcriptomic signatures for progression to TB disease.

**Methods:**

Close contacts (≥4 hours of exposure per week) of adult patients with culture-confirmed pulmonary TB were enrolled in Brazil. Investigation for incident, microbiologically confirmed, or clinically diagnosed pulmonary or extrapulmonary TB disease through 24 months of follow-up was symptom triggered. Twenty previously validated blood TB transcriptomic signatures were measured at baseline by real-time quantitative polymerase chain reaction. Prognostic performance for incident TB was tested by receiver operating characteristic curve analysis at 6, 9, 12, and 24 months of follow-up.

**Results:**

Between June 2015 and June 2019, 1854 close contacts were enrolled. Twenty-five progressed to incident TB, of whom 13 had microbiologically confirmed disease. Baseline transcriptomic signature scores were measured in 1789 close contacts. Prognostic performance for all signatures was best within 6 months of diagnosis. Seven signatures (Gliddon4, Suliman4, Roe3, Roe1, Penn-Nicholson6, Francisco2, and Rajan5) met the minimum World Health Organization target product profile for a prognostic test through 6 months and 3 signatures (Gliddon4, Rajan5, and Duffy9) through 9 months. None met the target product profile threshold through ≥12 months of follow-up.

**Conclusions:**

Blood transcriptomic signatures may be useful for predicting TB risk within 9 months of measurement among TB-exposed contacts to target preventive therapy administration.

## Teaser

Host blood transcriptomic signatures predicted progression to incident tuberculosis within 6 to 9 months of measurement among tuberculosis-exposed contacts and may be useful to target preventive therapy to those at greatest risk of progression, reducing unnecessary treatment of those at low risk.

An estimated 1.7 billion individuals have been infected with *Mycobacterium tuberculosis* (Mtb) [1]; however, <1% (approximately 10 million) progress to tuberculosis (TB) disease annually [2]. The risk of TB varies widely, with many persons with infection at low risk [3, 4]. Close contacts of individuals with TB are at relatively higher risk of developing TB, but 90% to 95% of such persons do not develop TB [5–7]. Efficacious short-course TB preventive therapy (TPT) regimens can prevent progression [8–12] but should focus on persons at highest risk of progression to TB. Risk of TB progression is highest in the first 1 to 2 years following exposure [13–15]. Although several clinical factors increase TB risk (eg, HIV infection and other immunocompromising conditions), there is a need for a short-term predictive test to identify those at highest risk of progression to active TB disease for targeted TPT [16]. Such a test would reduce unnecessary treatment of those at low risk of progression to TB.

Current tests for Mtb infection in clinical practice—the interferon-γ release assay (IGRA) and the tuberculin skin test (TST)—detect prior T-cell sensitization to Mtb antigens. While persons with a positive IGRA or TST result are at substantially increased risk of incident TB as compared with persons with a negative result [7, 17], the tests are unable to differentiate between cleared and persistent Mtb infection or between Mtb infection and TB disease. Consequently, these tests have very low positive predictive values (range, 2.2–4.2) for identifying those who will progress to active TB disease [7], with resultant overtreatment. IGRA and TST are also insufficiently sensitive prognostic tests for identifying progressors, missing incident TB cases at rates between 17% (TST, 5-mm threshold) and 39% (QuantiFERON-TB Gold In-Tube [QFT]; Qiagen) [7]. There are currently no tests for monitoring responses to TPT.

Host blood transcriptomic signatures show promise for identification of persons who will develop incident TB within 6 to 12 months of testing; however, most prognostic studies have been conducted in the United Kingdom and sub-Saharan Africa, limiting geographic generalizability [18–22]. Transcriptomic signatures have not been evaluated extensively in close TB contacts, who are at increased risk of progressing to TB within 1 to 2 years of TB exposure [5, 6]. We tested 20 published and previously validated host blood transcriptomic signatures for predicting progression to incident TB disease over 2 years of follow-up in a large multicenter cohort of close contacts of culture-confirmed pulmonary TB patients in Brazil. We also explored the association between isoniazid preventive therapy (IPT) and other participant characteristics, with transcriptomic signature scores among close contacts who did not develop incident TB during follow-up (nonprogressors).

## METHODS

### Study Design and Participants

Regional Prospective Observational Research for Tuberculosis (RePORT)–Brazil is a prospective observational cohort study that enrolled participants at 5 centers in Brazil: 3 in Rio de Janeiro (Instituto Nacional de Infectologia, Clínica da Família Rinaldo Delamare, and Secretaria de Saude de Duque de Caxias), 1 in Salvador (Instituto Brasileiro para Investigação da Tuberculose), and 1 in Manaus (Fundação Medicina Tropical Dr Heitor Vieira Dourado). The study protocol was approved by institutional research ethics committees at each participating site and collaborating institute (supplementary methods). Details of the protocol have been published previously [23]. Briefly, RePORT–Brazil enrolled adults (≥18 years) initiating treatment for culture-confirmed pulmonary TB and their close contacts. Close contacts were defined as individuals of any age with at least 4 hours per week of exposure to the source case prior to TB diagnosis, who were followed for up to 24 months for progression to incident TB disease. Close contacts were offered treatment with 6-month standard-of-care IPT. Those who discontinued follow-up prior to 24 months and did not meet the primary end point definition were censored at their final study visit but included in the prognostic analysis.

### TB Investigations and End Points

Investigation for TB disease among close contacts at enrollment and through the 24 months of follow-up was symptom triggered (eg, cough, fever, night sweats, loss of weight for ≥2 weeks, or hemoptysis) and included chest radiograph and sputum smear and culture. Symptomatic contacts with microbiologically confirmed prevalent TB at enrollment were excluded. The primary study end point was incident, pulmonary, or extrapulmonary TB disease as determined by local site investigators; participants with microbiologically confirmed TB had at least 1 sputum or extrapulmonary specimen positive for Mtb on culture. Clinical TB included those with clinical signs and symptoms of TB, with or without pathology consistent with TB (eg, necrotizing or caseating granulomas). Individuals diagnosed with TB disease were prescribed anti-TB chemotherapy per local guidelines.

### IGRA Measurement

Venous blood was collected for the QFT at enrollment and repeated at the month 6 visit in participants who were QFT negative at baseline. QFT interferon-γ responses ≥0.35 IU/mL were considered positive.

### Blood Sample Collection and RNA Extraction

Venous blood was collected in PAXgene RNA tubes (PreAnalytiX) at enrollment and month 6 visits for all participants, frozen at −20 °C, and shipped to the Instituto Gonçalo Moniz (Fundação Oswaldo Cruz). Samples were subsequently thawed, and RNA was manually extracted with the PAXgene Blood RNA Kit (Qiagen) according to the manufacturer's instructions. Approximately 50 ng of RNA per sample (∼8.3 ng/µL in 6 µL of water) was plated in 96-well polymerase chain reaction plates, frozen at −80 °C, and shipped to the laboratory of the South African Tuberculosis Vaccine Initiative (a member of the RePORT–South Africa Consortium).

### Transcriptomic Signature Panel Design and Measurement

Published TB transcriptomic signatures were selected by diagnostic and prognostic performance in recently published systematic reviews and head-to-head comparisons of published RNA signatures [19, 20, 24–26], as well as ongoing work in the South African Tuberculosis Vaccine Initiative laboratory [27]. We included 20 parsimonious signatures (defined as those that used feature reduction during discovery) based on the number of transcripts (<30), availability of primer-probe sequences or predesigned TaqMan assays, and signatures that were evaluated in at least 1 external validation cohort (Table 1). The 20 signatures were translated to microfluidic multiplex real-time quantitative polymerase chain reaction, reparameterized, and measured as previously described (supplementary methods) with a panel of prequalified TaqMan gene expression primer-probe assays (Supplementary Table 1; Thermo Fisher Scientific) [22, 27].

**Table 1.** Characteristics of Host Blood Transcriptomic Signatures Included in the Analysis

Signature			Discovery Cohort		
Name^a^	Reference^b^	Model	Country	Age Group	Application
da Costa3	*Tuberculosis* (2015)	Random forest	Brazil	Adults	Diagnostic; TB vs LTBI, HC, and OD
de Araujo1 (NPC2)	*Front Microbiol* (2016)	Standardized expression	Brazil	Adults	Diagnostic; TB vs LTBI and HC
Duffy9 (10)^c^	*PLoS One* (2019)	Six-class multinomial random forest	South Africa, Malawi	Adults	Diagnostic, TB vs LTBI and OD
Francisco2	*J Infect* (2017)	Random forest	China	Adults	Diagnostic; TB vs OD and HC
Gjøen7	*Sci Rep* (2017)	LASSO regression	India	Children	Diagnostic; TB vs HC
Gliddon3	*Front Immunol* (2021)	Disease risk score	South Africa, Malawi	Adults	Diagnostic; TB vs LTBI
Gliddon4	*Front Immunol* (2021)	Disease risk score	South Africa, Malawi	Adults	Diagnostic; TB vs OD
Jacobsen3	*J Mol Med* (2007)	Linear discriminant analysis	Germany	Adults	Diagnostic; TB vs LTBI and HC
Kaforou22 (27)^d^	*PLoS Med* (2013)	Disease risk score	South Africa, Malawi	Adults	Diagnostic; TB vs LTBI
Maertzdorf4	*EMBO Mol Med* (2016)	_RawCT_ID3 – [(_RawCT_GBP1 + _RawCT_IFITM3 + _RawCT_P2RY14)/3] (adapted from Suliman et al [37])	India	Adults	Diagnostic; TB vs HC and LTBI
Penn-Nicholson6	*Sci Rep* (2020)	Pairwise ensemble structure	South Africa	Adolescents	Prognostic; TB progressors vs nonprogressors
Rajan5	*Clin Infect Dis* (2019)	Unsigned sums	Uganda	Adults	Diagnostic; HIV+ TB vs HIV+ HC
Roe1 (BATF2)	*JCI Insight* (2016)	Standardized expression	UK	Adults	Diagnostic; TB vs HC and TB 2–4 y postrecovery
Roe3	*Clin Infect Dis* (2020)	SVM (linear kernel)	UK	Adults	Prognostic; TB progressors vs nonprogressors
Sambarey10	*EBioMedicine* (2017)	Linear discriminant analysis	India	Adults	Diagnostic; TB vs HC and LTBI
Satproedprai7	*Genes Immun* (2015)	LASSO regression	Thailand	Adults	Diagnostic; TB vs HC and previous TB
Suliman2	*Am J Respir Crit Care Med* (2018)	Pairwise ensemble structure	The Gambia, South Africa	Adults	Prognostic; TB progressors vs nonprogressors
Suliman4	*Am J Respir Crit Care Med* (2018)	Pairwise ensemble structure	The Gambia, South Africa	Adults	Prognostic; TB progressors vs nonprogressors
Sweeney3	*Lancet Respir Med* (2016); *JAMA Netw Open* (2018)	(_RawCT_GBP5 + _RawCT_DUSP3)/2 – _RawCT_KLF2	France, Malawi, South Africa, UK, USA	Adults	Diagnostic; TB vs HC + LTBI and OD
Thompson5	*Tuberculosis* (2017)	Pairwise ensemble structure	South Africa	Adults	Monitoring TB treatment response

Abbreviations: HC, healthy controls; LASSO, least absolute shrinkage and selection operator; LTBI, latent tuberculosis infection; OD, other diseases; SVM, support vector machines; TB, tuberculosis.

^a^Signatures are named by first author and number of transcripts included in the model (eg, Author11). Numbers in parentheses indicate the original number of transcripts in the published model. Some signatures have a reduced number of transcripts when translated to real-time quantitative polymerase chain reaction due to duplicate transcript symbols (IDs), high primer-probe failure rate (poor amplification efficiency), or where transcript sequences from original discovery cohorts could not be mapped to a more recent reference transcriptome.

^b^Full references are in the supplement.

^c^The CERKL1 primer-probe assay failed during panel optimization and was removed from the Duffy10 signature.

^d^Three transcripts (C1QB, C1QC, and GBP6) were excluded from the Kaforou27 signature due to a high primer-probe assay failure rate. Unique primer-probe assays could not be designed for both FCGR1B transcript variants; only 1 FCGR1B primer-probe was included in the Kaforou22 model. We were unable to design a primer-probe for LOC728744; all information had been withdrawn from the NCBI and RefSeq databases.

### Statistical Analysis

All analyses were performed with R Statistical Software (version 4.2.1; R Core Team 2022). Sample size was calculated per an estimate that 5% to 10% of persons with Mtb infection who did not receive IPT would progress to TB disease.

Signature score distribution was described by median and IQR. Differences between groups were calculated by the Mann-Whitney *U* test. Spearman rank coefficient was used to report correlation between signature scores; only samples with all assays passing were included in the correlation matrices.

For evaluation of prognostic performance, the receiver operating characteristic area under the curve (AUC) was generated by the pROC package in R [28]. AUC 95% CIs and comparison of AUCs were calculated with methods described by DeLong et al [29] and corrected for multiple comparisons by use of the Benjamini-Hochberg procedure [30]. Signature prognostic accuracy was benchmarked against the minimal criteria (sensitivity, 75%; specificity, 75%) and optimal criteria (sensitivity, 90%; specificity, 90%) as defined in the World Health Organization (WHO) target product profile (TPP) for a test to predict progression to incident TB disease [16]. Sensitivity, specificity, positive predictive value, and negative predictive value were calculated by binary end point indicators and standard formulae. The Wilson binomial proportion confidence interval formula was used for calculating 95% CIs for performance metrics. An alpha <.05 was considered statistically significant for all analyses.

## RESULTS

### Recruitment of TB Close Contact Cohorts

**Figure 1.**
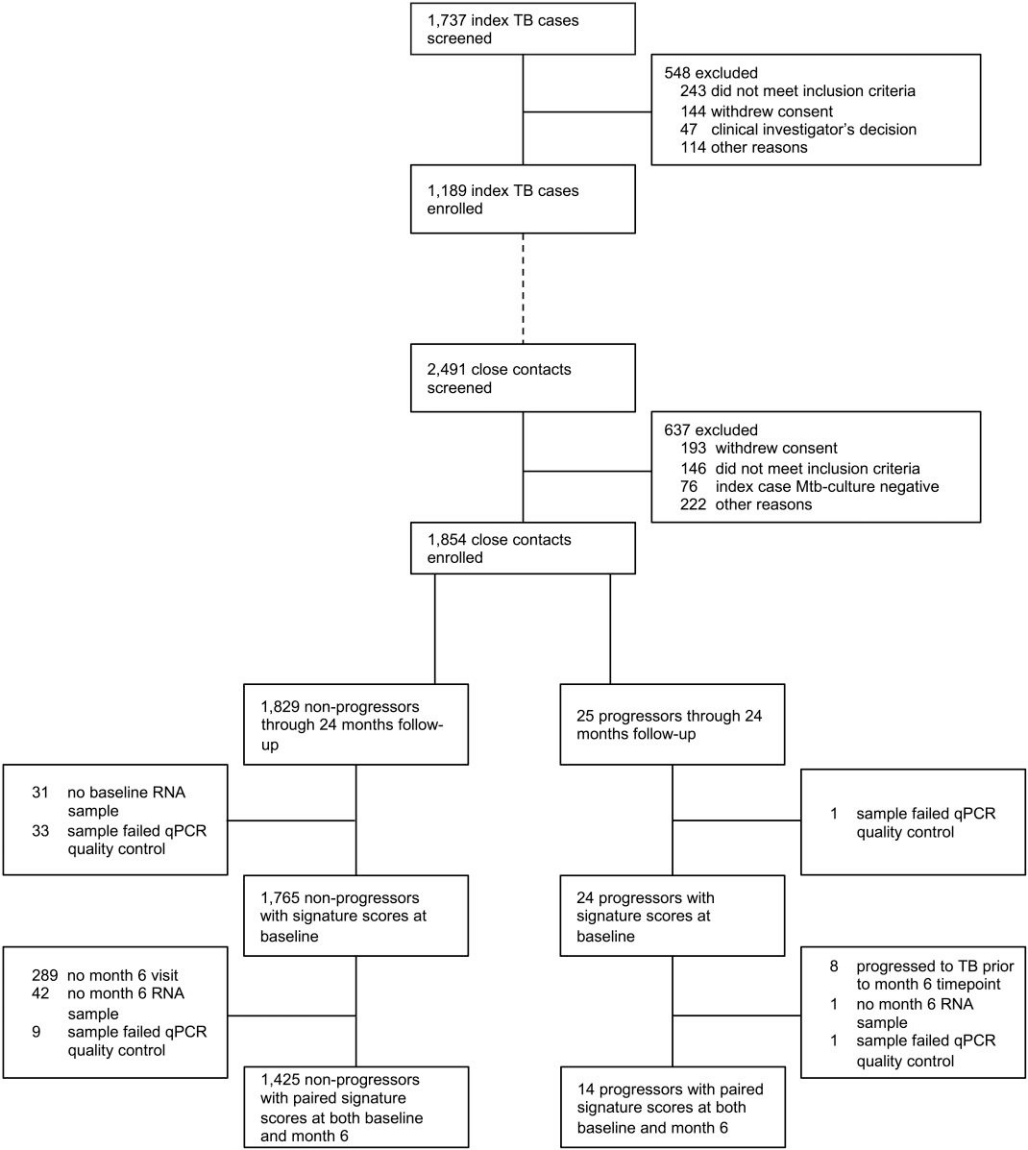
Study flow diagram. Mtb, *Mycobacterium tuberculosis*; qPCR, quantitative polymerase chain reaction; TB, tuberculosis.

Between June 2015 and June 2019, 1737 index TB cases were screened and 1189 enrolled (68%; Figure 1). The main reasons for exclusion of index cases included not meeting the inclusion criteria (n = 243, 44%) and withdrawal of consent (n = 144, 26%), as well as other reasons (n = 161, 29%), such as not being able to provide a sputum sample or not attending the entry visit. During the same period, 2491 close contacts of the enrolled index TB cases were screened and 1854 enrolled (74%). Close contacts were predominantly excluded for not meeting the inclusion criteria (n = 146, 23%) and withdrawal of consent (n = 193, 30%), as well as other reasons (n = 298, 47%), such as negative Mtb culture for the index TB case or not attending the entry visit. Of the 1854 enrolled close contacts, 684 (37%) were Mtb sensitized (IGRA positive) at baseline, and 12% (113/942) of baseline IGRA-negative contacts with subsequent testing at the month 6 visit converted to IGRA positive. Twenty-five (1.3%) contacts progressed to incident TB disease through 24 months of follow-up (progressors). Of 282 IGRA-positive close contacts who did not receive IPT, 15 (5.3%) developed TB through 24 months.

Of the 25 progressors, 19 (76%) had pulmonary TB and 6 (24%) extrapulmonary TB. Thirteen (52%) incident cases were microbiologically confirmed and 12 (48%) clinically diagnosed. Median time to progression was 10 months (IQR, 4–15). All 25 progressors were HIV seronegative. Progressors were more likely to have had a previous TB episode (*P* = .004) and a positive IGRA result at baseline (*P* < .001) than nonprogressors (Table 2 and Supplementary Table 2). Other demographic characteristics were similar between progressors and nonprogressors.

**Table 2.** Characteristics of the Close Contact Study Population

	No. (%) or Median (IQR)		
Characteristic	Nonprogressors (n = 1829)	Progressors (n = 25)	*P* Value^a^
Female	1080 (59.1)	16 (64.0)	.62
Missing	2	0	
Age, y	32.0 (16.1–46.4)	31.9 (18.3–50.1)	.39
Missing	2	0	
City			**.004**
Manaus	861 (47.3)	4 (16.0)	
Rio de Janeiro	667 (36.6)	14 (56.0)	
Salvador	294 (16.1)	7 (28.0)	
Missing	7	0	
Ethnicity			.55
Pardo	1085 (59.4)	12 (48.0)	
Black	365 (20.0)	7 (28.0)	
White	357 (19.6)	6 (24.0)	
Indian	14 (0.8)	0 (0.0)	
Asian	5 (0.3)	0 (0.0)	
Missing	3	0	
Smoking history			.94
Never	1341 (73.4)	20 (80.0)	
Former	298 (16.3)	3 (12.0)	
Current	187 (10.2)	2 (8.0)	
Missing	3	0	
Alcohol consumption history			.25
Never	846 (46.3)	16 (64.0)	
Former	353 (19.3)	3 (12.0)	
Current	627 (34.3)	6 (24.0)	
Missing	3	0	
Body mass index, kg/m^2^	24.3 (20.2–28.6)	24.2 (19.6–25.6)	.44
Missing	3	0	
HIV			
Positive	47 (2.6)	0 (0.0)	NA
Plasma viral load			NA
Undetectable (<50 copies/mL)	14 (46.7)	0 (NA)	
Detectable (>50 copies/mL)	16 (53.3)	0 (NA)	
Missing	17	0	
Previous TB	75 (4.1)	5 (20.0)	.**004**
Missing	8	0	
Baseline IGRA result			**<**.**001**
Negative	1127 (61.8)	5 (20.8)	
Positive	665 (36.5)	19 (79.2)	
Indeterminate	31 (1.7)	0 (0.0)	
Missing	6	1	
Baseline IGRA response, IU/mL	0.1 (0.0–1.2)	1.9 (0.4–5.9)	**<**.**001**
Missing	37	1	
Month 6 IGRA status^b^			.**001**
Persistent negative	825 (45.1)	4 (16.0)	
Converter (baseline negative)	112 (6.1)	1 (4.0)	
Baseline positive	665 (36.4)	19 (76.0)	
Missing	227 (12.4)	1 (4.0)	
IPT			.44
Receiving IPT at enrollment	10 (0.5)	0 (0.0)	
Started IPT during study	458 (25.0)	4 (16.0)	
No IPT received^c^	1361 (74.4)	21 (84.0)	
Completed IPT during study			.051
Completed 168 d (6 × 28)	323/468 (69.2)	1/4 (25.0)	
Completed <168 d (6 × 28)	121/468 (25.9)	2/4 (50.0)	
No IPT end date^d^	23/468 (4.9)	1/4 (25.0)	
No IPT received^c^	1362	21	
IPT received during study,^e^ d	187.0 (155.0–247.0)	48.0 (34.5–173.0)	.34
Completed IPT by month 6			.16
Completed 168 d (6 × 28)	216/468 (46.3)	1/4 (25.0)	
Completed <168 d (6 × 28)	71/468 (15.2)	0/4 (0.0)	
Started IPT after second PAXgene draw	53/468 (11.3)	0/4 (0.0)	
No repeat PAXgene collected	104/468 (22.3)	2/4 (50.0)	
No IPT end date^d^	23/468 (4.9)	1/4 (25.0)	
No IPT received^c^	1362	21	

Abbreviations: IGRA, interferon-γ release assay; IPT, isoniazid preventive therapy; NA, not applicable; TB, tuberculosis.

^a^Pearson chi-square test, Wilcoxon rank sum test, and Fisher exact test. Bold indicates significance.

^b^Repeat IGRA testing was performed at month 6 of follow-up only among participants who were IGRA negative at baseline (<0.35 IU/mL). Participants with baseline and month 6 negative IGRA results are termed “persistent negative.” “Converters” are those whose IGRA results are negative at baseline but convert to positive (≥0.35 IU/mL) at the month 6 visit.

^c^No IPT recorded as having been received during the study.

^d^Started IPT during study but with no treatment end date recorded during the study.

^e^Includes only participants with recorded IPT start and end dates.

### Transcriptomic Signatures Predicted Progression to TB Disease Through 9 Months of Follow-up

**Figure 2.**
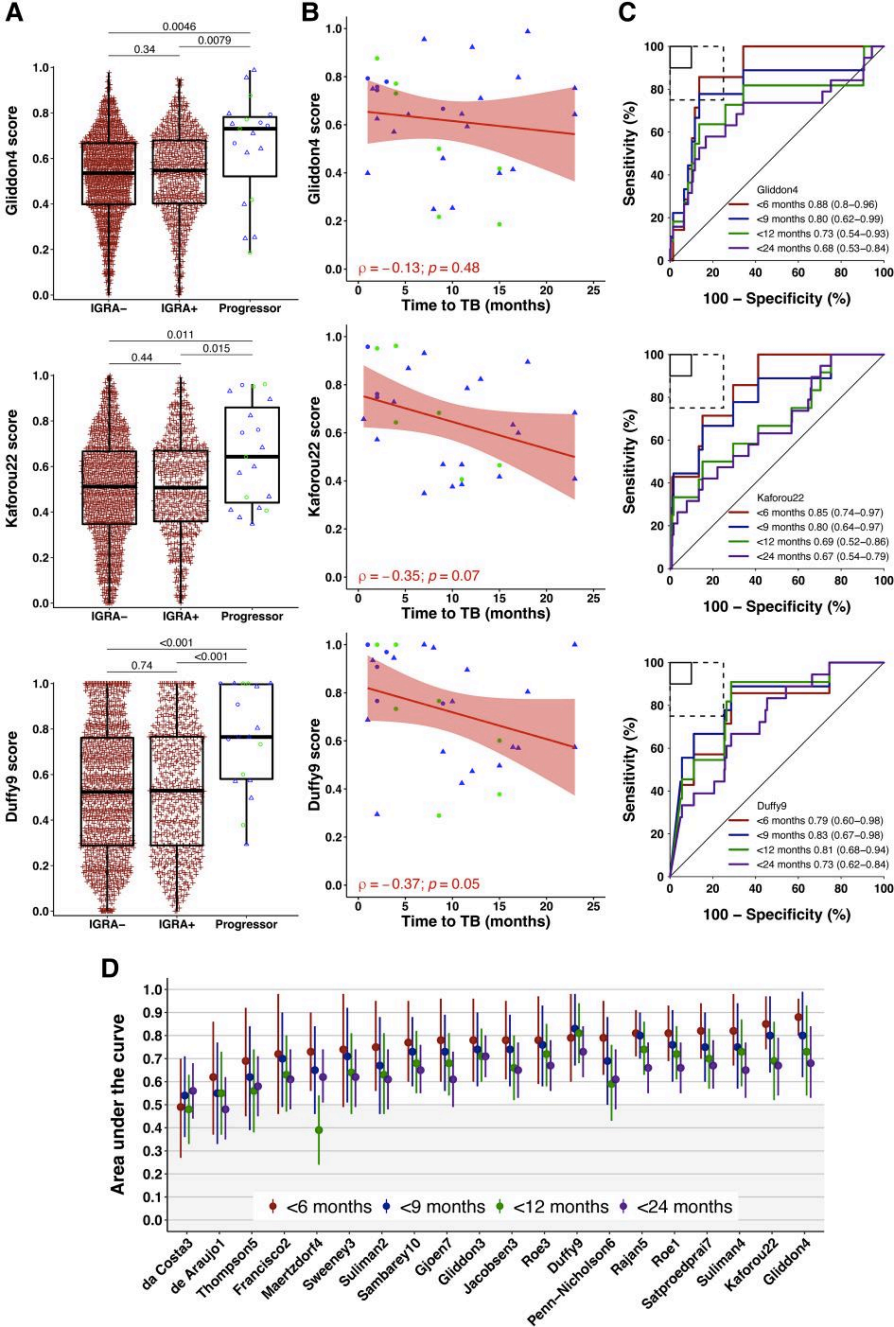
Prognostic performance of transcriptomic signatures. Representative (*A*) box-and-whisker plots, (*B*) scatter plots, and (*C*) ROC curves of the transcriptomic signatures with the best prognostic performance through 6 months (Gliddon4 and Kaforou22) and 9 through 24 months (Duffy9) of follow-up. *A*, The box-and-whisker plots depict signature score distribution at enrollment by TB status; each dot represents a participant. Red crosses represent IGRA-negative (IGRA−) and IGRA-positive (IGRA+) nonprogressors; blue and green dots, pulmonary and extrapulmonary incident TB cases, respectively; and triangles and circles, microbiologically confirmed and clinically diagnosed TB, respectively. *P* values for comparison of median signature scores between groups in the box-and-whisker plots were calculated with the Mann-Whitney *U* test. Box, IQR; midline, median; whiskers, IQR ± (1.5 × IQR). Numbers of participants are included in Table 3. *B*, The scatter plots depict signature score distribution at enrollment and month 6 of follow-up by months to incident TB diagnosis. Each dot represents a participant with incident TB; blue and green dots, pulmonary and extrapulmonary TB cases, respectively; and triangles and circles, microbiologically confirmed and clinically diagnosed TB, respectively. LOESS curve, the local polynomial regression; shaded area, 95% CI on the LOESS regression. Spearman rank order correlation coefficient (ρ), demonstrating the association between time to incident TB diagnosis and signature score, and *P* values are shown for each signature. *C*, The ROC curves depict the prognostic performance (AUC with 95% CI) of signatures for differentiating progressors (incident TB disease) vs nonprogressors (healthy close contacts) through 6, 9, 12, and 24 months of follow-up. Numbers of participants are included in Table 3. The solid box depicts the optimal criteria (90%, sensitivity; 90%, specificity) and the dashed box the minimal criteria (75%, sensitivity; 75%, specificity) as set out in the World Health Organization’s target product profile for an incipient TB test [16]. (*D*) Summary of signature prognostic performance in the order of AUC estimates through 6 months of follow-up. The prognostic AUC estimates through 9, 12, and 24 months are also shown. Midline, the AUC estimate; error bars, 95% CI. AUC, area under the curve; IGRA, interferon-γ release assay; LOESS, locally estimated scatterplot smoothing; ROC, receiver operating characteristic; TB, tuberculosis.

Transcriptomic signature scores were measured in 1789 (96%) close contacts at baseline (analysis population), including 24 (1.3%) progressors (Figure 1). As scores for signatures were highly correlated (Supplementary Figure 1), Gliddon4, Kaforou22, and Duffy9 signatures were selected post hoc for display in all figures as the best-performing signatures for incident TB prognosis through 6 months (Gliddon4 and Kaforou22) and 9 through 24 months (Duffy9) of follow-up. Transcriptomic signature scores at baseline were similar among IGRA-positive and IGRA-negative close contacts for most signatures, with small differences observed for some signatures (Figure 2*A*; other signature data not shown). Yet, scores were significantly higher among baseline samples for the 24 progressors than the IGRA-positive or IGRA-negative nonprogressors for 17 of 20 signatures, with a trend toward higher scores proximal to TB diagnosis for most signatures (Figure 2*B*; other signature data not shown). Consequently, better signature prognostic performance was observed at time intervals most proximal to TB diagnosis (eg, within 6 months of measurement) and waned for most signatures after 9 months of follow-up (Figure 2*C* and 2*D*). Signature scores for individuals with microbiologically confirmed TB disease (n = 12; 1 individual missing signature scores) were not different to those of clinically diagnosed disease (n = 12); similarly, scores for pulmonary incident TB disease (n = 18) were not different to those for extrapulmonary disease (n = 6; Wilcoxon rank sum *P* > .05 for all signatures, data not shown). However, this analysis was limited by the small number of progressors in these groups.

Gliddon4 had the highest AUC point estimates through 6 months of follow-up (AUC, 0.88; 95% CI, .80–.96), while Duffy9 had the highest AUC point estimates through 9 months (AUC, 0.83; 95% CI, .67–.98), 12 months (AUC, 0.81; 95% CI, .68–.94), and 24 months (AUC, 0.73; 95% CI, .62–.84) of follow-up (Figure 2*C* and 2*D*, Table 3). However, Gliddon4 and Duffy9 performance was not statistically different from 15 of the other signatures (Figure 2*D*, Supplementary Figure 2). The statistical power for these analyses was limited by the small numbers of progressors. Transcriptomic signatures did not outperform the QFT assay, which also had a trend toward superior performance proximal to incident TB diagnosis, with AUCs of 0.84 (95% CI, .75–.92), 0.79 (95% CI, .65–.94), 0.75 (95% CI, .63–.87), and 0.72 (95% CI, .62–.82) through 6, 9, 12, and 24 months of follow-up, respectively.

**Table 3.** Transcriptomic Signature Prognostic Performance Among All Study Participants With Transcriptomic Signature Scores at Enrollment

		TB Cases				AUC (95% CI)			
	Healthy Nonprogressors	6 mo	9 mo	12 mo	24 mo	6 mo	9 mo	12 mo	24 mo
QuantiFERON TB-Gold^a^	1730	8	11	15	24	0.84 (.75–.92)	0.79 (.65–.94)	0.75 (.63–.87)	0.72 (.62–.82)
Gliddon4	1530	7	9	11	19	0.88 (.80–.96)	0.80 (.62–.99)	0.73 (.54–.93)	0.68 (.53–.84)
Kaforou22	1334	7	9	12	19	0.85 (.74–.97)	0.80 (.64–.97)	0.69 (.52–.86)	0.67 (.54–.79)
Suliman4	1757	8	11	15	24	0.82 (.67–.98)	0.75 (.57–.94)	0.73 (.58–.87)	0.65 (.53–.77)
Satproedprai7	1662	8	11	15	24	0.82 (.70–.94)	0.75 (.60–.90)	0.70 (.57–.83)	0.67 (.57–.78)
Rajan5	1537	7	9	11	18	0.81 (.71–.91)	0.80 (.70–.90)	0.74 (.63–.86)	0.66 (.55–.77)
Roe1	1744	8	10	14	23	0.81 (.69–.93)	0.76 (.60–.91)	0.72 (.61–.84)	0.66 (.55–.77)
Penn-Nicholson6	1761	8	11	15	24	0.79 (.63–.95)	0.69 (.50–.88)	0.59 (.43–.76)	0.61 (.48–.74)
Duffy9	1400	7	9	11	18	0.79 (.60–.98)	0.83 (.67–.98)	0.81 (.68–.94)	0.73 (.62–.84)
Gliddon3	1594	7	10	14	22	0.78 (.60–.96)	0.74 (.58–.90)	0.71 (.60–.83)	0.71 (.62–.80)
Jacobsen3	1748	8	11	15	24	0.78 (.61–.95)	0.74 (.58–.89)	0.66 (.52–.80)	0.65 (.53–.77)
Roe3	1743	8	10	14	23	0.78 (.59–.97)	0.76 (.58–.93)	0.72 (.58–.85)	0.67 (.56–.78)
Gjoen7	1763	8	11	15	24	0.78 (.60–.96)	0.73 (.56–.89)	0.68 (.54–.81)	0.61 (.49–.73)
Sambarey10	1751	8	11	15	24	0.77 (.60–.95)	0.73 (.58–.88)	0.68 (.55–.82)	0.65 (.55–.76)
Suliman2	1750	8	11	15	24	0.75 (.56–.95)	0.67 (.46–.88)	0.63 (.46–.81)	0.61 (.48–.74)
Sweeney3	1759	8	11	15	24	0.74 (.49–.98)	0.71 (.51–.92)	0.64 (.46–.81)	0.62 (.49–.74)
Maertzdorf4	1703	8	11	15	24	0.73 (.56–.90)	0.65 (.46–.84)	0.39 (.24–.54)	0.62 (.51–.74)
Francisco2	1759	8	11	15	24	0.72 (.46–.98)	0.70 (.49–.90)	0.63 (.47–.80)	0.61 (.48–.74)
Thompson5	1764	8	11	15	24	0.69 (.45–.92)	0.62 (.39–.84)	0.56 (.38–.74)	0.58 (.45–.71)
de Araujo1	1761	8	11	15	24	0.62 (.37–.86)	0.55 (.33–.77)	0.55 (.37–.73)	0.48 (.35–.62)
da Costa3	1765	8	11	15	24	0.49 (.27–.70)	0.54 (.36–.71)	0.48 (.33–.63)	0.56 (.44–.68)

Transcriptomic signatures are sorted by prognostic performance (AUC) through 6 months of follow-up.

Abbreviations: AUC, area under the curve; TB, tuberculosis.

^a^QuantiFERON TB-Gold AUCs were calculated per quantitative results.

Gliddon4, Suliman4, Roe3, Roe1, Penn-Nicholson6, Francisco2, and Rajan5 met the minimum prognostic TPP criteria (sensitivity, 75%; specificity, 75%) through 6 months of follow-up (Supplementary Table 3A). Gliddon4, Rajan5, and Duffy9 met this threshold through 9 months of follow-up. Notably, only the Duffy9 signature approached this threshold through 12 months of follow-up (sensitivity, 81.8% [95% CI, 52.3%–94.9%]; specificity, 73.6% [95% CI, 71.2%–75.8%]). No signatures met the benchmark criteria through 24 months of follow-up. At the manufacturer’s positivity cutoff (≥0.35 IU/mL), QFT had a specificity of 62.8% (95% CI, 60.5%–65.0%) and sensitivities of 100% (95% CI, 67.6%–100%), 90.9% (95% CI, 62.3%–98.4%), 86.7% (95% CI, 62.1%–96.3%), and 79.2% (95% CI, 59.5%–90.8%) through 6, 9, 12, and 24 months of follow-up, respectively.

Receipt of IPT during the study may have affected the prognostic accuracy of the signatures. In a sensitivity analysis, we excluded progressors (4/24, 17%) and nonprogressors (449/1765, 25%) who were receiving IPT at the time of enrollment or received at least 1 dose of IPT during the study. TB incidence among participants who did not receive IPT was low: 0.4% (95% CI, .2%–1.0%), 0.7% (95% CI, .4%–1.3%), 1.0% (95% CI, .6%–1.7%), and 1.5% (95% CI, 1.0%–2.3%) through 6, 9, 12, or 24 months of follow-up, respectively (Supplementary Table 3B). When limited to participants who did not receive IPT, a similar pattern of prognostic performance was found as compared with the full cohort (Supplementary Figure 3A). Signature AUC, sensitivity, and specificity were not higher when participants who received IPT were excluded (Supplementary Table 3B–D). Transcriptomic signatures performed as well as IGRA through 9 months but had lower prognostic accuracy through 12 and 24 months.

## DISCUSSION

This study showed that blood transcriptomic signatures are capable of identifying close contacts of patients with TB who are at risk of incident TB within 9 months of testing. Seven signatures met the WHO TPP for sensitivity and specificity of TB progression within a 6-month period and 3 signatures within 9 months: Gliddon4, Duffy9, and Rajan5. The importance of identifying who will progress to TB within 6 to 12 months of TB exposure is supported by epidemiologic data from Reichler et al [15], demonstrating that the TB risk among close contacts is highest during this period; notably, 81% and 92% of incident TB cases occurred within 6 and 12 months, respectively, in a recent prospective close-contact cohort. Several additional studies showed that most TB in close contacts occurs in the first 12 months after exposure [31–33]. This is also important because contact investigations are conducted after TB exposure and preventive therapy is administered to close contacts at high risk of TB. Transcriptomic signatures may be useful for prediction of TB risk and targeted preventive therapy administration [21, 34, 35].

Our results in a Brazilian population are consistent with previous findings from studies conducted in Africa and the United Kingdom [18, 19, 21, 36, 37], supporting the geographic generalizability of the signatures. This suggests that signatures are not affected by population genetic differences. This study enrolled close contacts of index patients with TB, rather than healthy adults from the general population. In the CORTIS-01 trial, which recruited healthy adults who were not seeking care from TB-endemic communities in South Africa, the RISK11 11-gene TB prognostic signature was able to predict progression through 6 months, although performance waned through 12 months [21]. The finding in that study that incident cases occurring after 12 months had lower RISK11 scores at enrollment may have been due to reinfection during study follow-up or could have reflected early disease with minimal inflammatory activity at enrollment [38]. This finding was consistently observed for other parsimonious signatures in the South African CORTIS-01 populations [22], including people living with HIV [39], and is supported by a patient-level pooled meta-analysis, which revealed a 3- to 6-month transcriptomic signature prognostic window [19]. In this study, as well other cohorts reported in the literature [18, 19, 21, 36, 37], transcriptomic signatures do not meet the benchmark criteria for an incident TB test over the prognostic period stipulated in the WHO TPP (2 years) [16], and better specificity is warranted. However, the 2-year time frame is probably not appropriate, given that TB risk is highest within 6 to 12 months among close contacts [15], and we propose that this guidance be revisited. Genes represented by the parsimonious signatures measured in this cohort predominantly mapped to interferon-stimulated and complement pathways [20, 26, 40], which are typically the most differentially expressed genes between TB cases and controls in discovery cohorts. We did not perform extensive analyses of the represented biologically significant pathways because the panel of approximately 90 transcripts do not represent the breadth and depth of gene expression necessary for this.

The QFT assay performed as well or better than transcriptomic signatures for prognosis of incident pulmonary TB in the current Brazilian study. However, the specificities of several signatures were higher than QFT through 9 months; thus, measurement of signatures may be useful as a TPT sparing approach. Combination of QFT and transcriptomic signatures resulted in a several-fold-higher positive predictive value vs QFT alone, as previously shown [41]. For example, risk of incident TB among participants who were QFT positive and did not receive IPT was markedly higher through 6 and 9 months of follow-up among participants who were Gliddon4 signature positive than those who were Gliddon4 negative. A combined screening approach could be used to target those who would benefit most from receiving TPT among close contacts but would be unlikely to be cost-effective or practicable outside of a research setting. In the CORTIS-01 trial, transcriptomic signatures outperformed the QFT assay through 12 months of follow-up among individuals with or without HIV [21, 39]. This result may suggest that transcriptomic signatures could have a role in guiding TPT in settings with higher TB incidence, where the majority of the general population is QFT positive and QFT therefore has lower specificity for recent Mtb infection. Conversely, QFT may have greater specificity for recent discrete exposure among close contacts in low-transmission settings.

A key question is whether transcriptomic signatures have utility for monitoring host response to TPT. Prior TPT treatment has been associated with lower blood transcriptomic signature scores among people with HIV [42], and anti-TB treatment of suspected Mtb infection has been shown to downregulate expression of signatures of TB risk [43]. However, the current study supports the hypothesis that TPT does not have an effect on transcriptomic signature scores. Similarly, changes in RISK11 signature scores could not be attributed to 3HP (3 months of weekly rifapentine and isoniazid) in the CORTIS-01 study [44]. The scores in 74.4% of 3HP recipients (who were all RISK11 positive at randomization) significantly decreased, but so did the scores in 73.1% in the RISK11-positive untreated arm, suggesting that the decrease in scores was unrelated to TPT. The transient nature of interferon-stimulated gene signature positivity may reflect intercurrent upper respiratory viral infection, highlighting the challenges of implementing such signatures for risk of progression to incident TB disease and monitoring response to TPT.

Scores for most signatures correlated strongly, suggesting that the majority of transcriptomic signatures measure a common biological signal [22]. The majority of the included signatures were developed for TB diagnosis rather than prediction of disease progression. Yet, Tabone et al [45] and Scriba et al [46] showed that similar gene modules—predominantly, interferon-stimulated and complement pathway genes—are differentially expressed in incipient, subclinical, and clinical TB disease but with the greatest fold change in expression in clinical TB, a weaker signal in subclinical TB, and the weakest in early/incipient TB (furthest from progression). This supports the hypothesis that TB transcriptomic signature scores reflect the spectrum of TB disease, with the highest scores proximal to TB diagnosis with resultant better short-term predictive performance. Future transcriptomic signature discovery should include filters to avoid dominance of a single module, such as the interferon-stimulated gene pathway, as done by Esmail et al [40, 47]. However, it is unlikely that yet more novel transcriptomic signatures will detect a stronger “signal” where other studies have failed and will substantively improve on the prognostic performance in this study or allow monitoring of response to TPT. New Mtb-specific biomarkers that are not induced by unrelated infectious organisms are needed.

This study had several limitations. First, while the cohort size of close contacts was large, a small number of participants (n = 25) progressed to incident TB disease, and early prognostic windows (ie, 6 or 9 months) had even smaller numbers of progressors. This resulted in wide confidence intervals for performance estimates. The number of incident cases was also too small to perform reliable subgroup analyses, such as extrapulmonary TB, and precluded training and testing a new ensemble model combining multiple genes or signatures. However, the number of progressors was as expected: approximately 5% of IGRA-positive close contacts who did not receive IPT. Second, not all progressors had microbiologically confirmed TB. Prior studies show that performance of transcriptomic signatures is poorer for discriminating probable vs microbiologically confirmed TB [27]. Third, IPT was not directly observed and adherence not monitored; thus, we are not able to reliably ascertain treatment completion and effect on the transcriptomic signatures. Fourth, evaluation for TB among close contacts was based on the presence of symptoms of TB; subclinical TB was not captured and such persons could have progressed to symptomatic TB. Fifth, study participants lost to follow-up could have developed TB, and such cases were not captured. While these are important limitations, they highlight the complexity that large clinical studies require to accrue sufficient numbers of incident cases to perform reliable studies of progression biomarkers. Despite this, it is critical that more such studies are conducted to advance the development of novel prognostic tools.

In conclusion, blood transcriptomic signatures may have utility for prediction of incident TB within 9 months of evaluation of close contacts of patients with TB. Three signatures met WHO TPP sensitivity and specificity over a 9-month period and identified close contacts at particularly increased TB risk. Further investigation is merited to evaluate the role of these signatures in targeted TPT and to reduce unnecessary treatment.

## Supplementary Data

Supplementary materials are available at *The Journal of Infectious Diseases* online (http://jid.oxfordjournals.org/). Supplementary materials consist of data provided by the author that are published to benefit the reader. The posted materials are not copyedited. The contents of all supplementary data are the sole responsibility of the authors. Questions or messages regarding errors should be addressed to the author.

## Supplementary Material

jiae237_Supplementary_Data

## Notes

***Author contributions*.** B. B. A., A. P.-N., M. H., T. R. S., and T. J. S. conceived the idea. B. B. A., A. P.-N., T. R. S., and T. J. S. raised funds and/or provided the resources. V. C. R., M. C.-S., and A. L. K. were responsible for all site-level activities, including recruitment, clinical management, and data collection. S. C. M., S. K. M., A. M. S. A., and M. C. F. provided operational, technical, laboratory support, or project management. V. M. M. and M. E. processed samples and performed the experiments. S. C. M., P. K. T., and M. C. F. verified the underlying data. S. C. M. and P. K. T. analyzed the data. S. C. M., T. R. S., and T. J. S. interpreted the results and wrote the first draft. All authors had full access to the data; confirm the integrity of the data and its presentation; agree with its interpretation as discussed in the article; and reviewed, revised, and approved the manuscript before submission. The corresponding author had final responsibility for the decision to submit for publication.

***Data availability statement.*** Deidentified quantitative polymerase chain reaction data, signature scores, and TB end point data underlying the results have been deposited in Zivahub (https://doi.org/10.25375/uct.24942711), an open access data repository hosted by the University of Cape Town's institutional data repository powered by Figshare for Institutions. Additional clinical metadata are available on request from the RePORT–Brazil Consortium.

***Disclaimer.*** The funders had no role in study design, data collection and analysis, decision to publish, or preparation of the manuscript.

***Study teams.*** RePORT–South Africa Consortium: Nicole Bilek, Yolundi Cloete, Mzwandile Erasmus, Michelle Fisher, Katie Hadley, Rieyaat Hassiem, Mark Hatherill, Lungisa Jaxa, Stanley Kimbung Mbandi, Simon C. Mendelsohn, Faheemah Meyer, Vanessa M. Muwanga, Onke Nombida, Adam Penn-Nicholson, Rodney Raphela, Thomas J. Scriba, Alison September, Timothy R. Sterling, Prisca K. Thami, Ashley Veldsman. RePORT–Brazil Consortium: Alice Andrade, Bruno B. Andrade, Brenda Carvalho, Marcelo Cordeiro-Santos, Marina Cruvinel Figueiredo, Adriano Gomes, Afranio L. Kritski, Valeria C. Rolla, Timothy R. Sterling.

***Financial support*.** This work was supported by the National Institutes of Health (U01 AI069923, U01 AI172064, R01 AI147765, R01 A1120790, F31 AI152614); CRDF Global (R-202208-69014); and Departamento de Ciência e Tecnologia, Secretaria de Ciência e Tecnologia, Ministério da Saúde, Brazil (25029.000507/2013-07).
