# Supplementary material for: Transcriptomic Signatures of Progression to Tuberculosis Disease Among Close Contacts in Brazil
Source: J Infect Dis. 2024 May 6;230(6):e1355–65. doi: 10.1093/infdis/jiae237 (PMC11646616; doi:10.1093/infdis/jiae237)
Supplement: jiae237_Supplementary_Data [file jiae237_supplementary_data.pdf]

Supplement to:

Transcriptomic signatures of progression to TB disease among close contacts in Brazil

Simon C Mendelsohn,[‡#1] Bruno B Andrade,[#2] Stanley Kimbung Mbandi,[1] Alice MS Andrade,[2] Vanessa M Muwanga,[1] Marina C Figueiredo,[3] Mzwandile Erasmus,[1] Valeria C Rolla,[4] Prisca K Thami,[1] Marcelo Cordeiro-Santos,[5] Adam Penn-Nicholson,[1,6] Afranio L Kritski,[7] Mark Hatherill,[1] Timothy R Sterling,[#3] Thomas J Scriba,[#1] and the RePORT-South Africa and RePORT-Brazil Consortia

Author affiliations:

[1]South African Tuberculosis Vaccine Initiative, Institute of Infectious Disease and Molecular Medicine and Division of Immunology, Department of Pathology, University of Cape Town, Anzio Road, Observatory, 7935, Cape Town, South Africa.

[2]Laboratório de Pesquisa Clínica e Translacional, Instituto Gonçalo Moniz, Fundação Oswaldo Cruz, Salvador, Bahia 40296-710, Brazil.

[3]Vanderbilt University Medical Center, Nashville, TN 37232, USA.

[4]Laboratorio de Pesquisa Clinica em Micobacterioses, Instituto Nacional de Infectologia Evandro Chagas, Fiocruz, Rio de Janeiro 21040-360, Brazil.

[5]Fundação de Medicina Tropical Doutor Heitor Vieira Dourado.

[6]FIND, 9 Chemin des Mines, 1202, Geneva, Switzerland.

[7]Universidade Federal do Rio de Janeiro, Rua Prof Rodolpho Paulo Rocco, 255, Centro de Pesquisa em Tuberculose, Cidade Universitária, 21941-913, Rio de Janeiro, RJ, Brazil.

[‡]Corresponding author: Simon C Mendelsohn

Email: Simon.Mendelsohn@uct.ac.za **Telephone:** +27 (0)21 406 6791

Postal address: South African Tuberculosis Vaccine Initiative, Institute of Infectious Disease and Molecular Medicine and Division of Immunology, Department of Pathology, University of Cape Town, Anzio Road, Observatory, 7935, Cape Town, South Africa.

[#]Contributed equally

Contents

Supplementary Methods 3
Supplementary Results 6
Supplementary Discussion 7
Supplementary Tables 8
Supplementary Figures 18
Supplementary References 27

Supplementary Methods

Ethical approval

The study protocol was approved by the ethics committee of the Maternidade Climério de Oliveira, Salvador, Brazil, the institutional research ethics committees at each participating site, and Vanderbilt University Medical Center. The University of Cape Town Faculty of Health Sciences Human Research Ethics Committee (HREC 594/2018) approved the analysis of transcriptomic signatures on all samples collected. All participants provided written, informed consent; all research was performed in accordance with relevant guidelines/regulations; and all experiments conformed to the principles set out in the WMA Declaration of Helsinki and the US Department of Health and Human Services Belmont Report.

Transcriptomic signature panel design and measurement

RNA plates were thawed in batches, cDNA synthesised with EpiScript reverse transcriptase (Lucigen, Middleton, WI, USA), and genes of interest pre-amplified using pools of TaqMan primer-probe assays (**Table S1**). Gene expression (raw cycle threshold, Ct) was quantified by microfluidic multiplex RT-qPCR using Standard BioTools (formerly Fluidigm, San Francisco, CA, USA) 96.96 (96 samples multiplexed with 96 primer-probe assays) Gene Expression chips on a BioMark HD instrument (Standard BioTools). Quality control filters, batch correction, and calculation of the twenty signature scores was performed using a pre-defined R script.

Data quality control

Standard BioTools 96.96 gene expression data were analysed using a pre-defined R script with quality control filters that assessed the integrity and reproducibility of each chip. The following parameters were applied for extracting Ct values: Linear (Derivative) baseline correction, Quality Threshold of 0.3, and Auto (Global) for Ct Threshold Method using Standard BioTools Biomark software version 4.5.2. No-template (water) and internal positive control samples were run on each chip. Chips with marked deviation (>5 standard deviations, Spearman correlation < 0.98, or concordance correlation coefficient < 0.95) in the internal positive control sample primer-probe assay raw Ct values or Penn-Nicholson6 (RISK6) signature score versus 11 historical runs, with amplification (raw Ct < 35 cycles) for any assay in the no-template control, or with more than 10% failed primer-probes, were repeated. Individual samples with more than 20% failed primer-probe reactions were classified as failed and no signature scores were computed. If less than 20% of primer-probe reactions failed for an individual sample, signature scores were computed where possible and signatures with missing primer-probe raw Ct values were deemed failed for that sample. Samples and primer-probe assays were run in singlet, and failed signature results for individual samples were assumed to follow a random distribution, thus not repeated, and excluded from analysis.

Signature score calculation

Signature scores were calculated from raw Ct measurements using the original algorithms developed for each individual signature, where possible (**Table 1**).[1-19] Signatures discovered using

methods other than RT-qPCR, such as RNA-seq or microarray, were subsequently re-parameterised to RT-qPCR.[20-22] In cases where predesigned TaqMan primer-probe assays were not available from the original publication, we used either inventoried primer-probes or designed novel TaqMan assays based on the published Illumina probe, RNA-seq transcript, or primer-probe sequence data. Furthermore, we contacted the authors of relevant publications when the required information was not available. We included three reference primer-probes for standardisation of gene expression for signatures which require a normalised (delta) cycle threshold (Ct).

Signature reparameterisation

Eight signatures (da Costa3, Duffy9, Francisco2, Gjøen7, Jacobsen3, Roe3, Sambarey10, and Satproedprai7) developed using gene expression analysis techniques other than real-time quantitative PCR (RT-qPCR) required reparameterisation in order to compute signature scores from microfluidic multiplex RT-qPCR BioMark HD readouts. This was necessary because different platforms each have unique dynamic ranges and quantitative scales for quantifying gene expression levels. The machine learning models, and statistical methods used for computing signature scores therefore needed to be reconstructed (reparameterised) using cycle threshold (Ct) values generated from RT-qPCR runs on the BioMark HD platform. This ensured that there was uniformity in the way scores were calculated for different signatures and how results were interpreted. The model parameters for the eight signatures[1,3-5,7,13-15] were reconstructed using the original published methods to best differentiate between TB cases and controls when adapted to Ct values, generated using PAXgene RNA samples from the previously described cross-sectional TB cohort (CTBC).[10,23] This cohort included both HIV positive and HIV negative TB cases and healthy controls. The use of this independent dataset for reparameterisation reduced potential bias. The RT-qPCR protocol for the reparameterisation experiments was consistent with the methods used for all RT-qPCR in this paper.

Model training was done on individuals that were HIV negative (59 LTBI; 50 TB). 70% of HIV negative individuals (42 LTBI; 34 TB) made up the training set and the remaining 30% comprised the test set (17 LTBI; 16 TB). Once a model constructed on the training set demonstrated discrimination on the test set, then both sets were combined to reparameterise on the entire dataset (**Table S5**). ROC curves were generated to validate the performance of each model's ability to separate HIV-positive TB cases (n=39) from HIV-positive LTBI controls (n=36) (**Table S5**).

A number of signatures had not previously been evaluated using the Biomark RT-qPCR instrument, but did not require reparameterisation even though they were discovered using approaches other than RT-qPCR. Examples include the Gliddon3/4,[6] Kaforou25,[8] and Rajan5[11] signatures. Gliddon3/4 and Kaforou25 were discovered using microarray data using a simple Disease Risk Score (DRS), which sums normalized expression values from all upregulated transcripts and subtracts the normalized expression values of all downregulated transcripts, making the signature essentially "self-normalizing". Rajan5 was also discovered based on microarray gene expression data, but developed using the signed and unsigned sums methods, which also allowed the signature to be "self-normalising".

Batch correction and score normalisation

Batch correction was performed to correct for differences in signature score distribution between Fluidigm chip runs using a quantile regression method with the *adjust_batch* function in the *batchtma* R package.[24] This method unifies lower quantiles (25th percentile) and ranges between the lower and upper quantile (75th percentile) between batches, allowing for differences in both parameters due to confounders. Thereafter, signature scores were winsorized (clipped) to account for extreme outliers (*Winsorize* function, *DescTools* R package[25]), log transformed to normalise score distribution (*log* function, *base* R package), converted to z-scores (*scale* function, *base* R package), and compared using the cumulative distribution function (*pnorm* function, *stats* R package). De Araujo1, Rajan5, and Roe1 signatures were multiplied by −1 to obtain a positive correlation for all signatures.

Multivariable regression analyses

Multivariable linear regression models were built to assess the effect of participant characteristics on signature scores among non-progressors, and results were corrected for multiple comparisons by use of the Benjamini–Hochberg Procedure.[26]

Blinding

Participants and study staff responsible for TB investigation in Brazil were blinded to transcriptomic signature scores. Transcriptomic signature scores were measured by laboratory personnel in South Africa who were blinded to participant TB status. Signature scores and TB microbiology results were maintained in different files, which were integrated only after the study database had been cleaned and locked.

Supplementary Results

Sensitivity analyses

Signature performance among baseline IGRA-positive participants (642 non-progressors and 19 progressors) was similar to that of the whole cohort (**Figure S3B**). When restricted to IGRA-positive individuals who did not receive IPT during the study (262 non-progressors and 15 progressors), AUC estimates were reduced for most signatures (**Figure S3C**), but showed wide confidence intervals. TB incidence among IGRA-positive participants who did not receive IPT was 2.2% (95%CI 1.0–4.8), 3.0% (95%CI 1.5–5.7), 4.0% (95%CI 2.3–7.1), and 5.4% (95%CI 3.3–8.7) through 6, 9, 12, or 24 months, respectively (**Table S3D**). If the Gliddon4 signature (with cut-off set at minimum WHO TPP threshold) was used to predict progression to TB disease among only IGRA-positive participants who did not receive IPT, the risk of incident TB (PPV) would be 12.9% (95%CI 5.1–28.9), 5.8% (95%CI 2.5–12.9), 2.9% (95%CI 1.3–6.1), and 4.2% (95%CI 2.2–7.9) through 6, 9, 12, or 24 months, respectively. Gliddon4 signature NPV among IGRA-positive participants who did not receive IPT were 99.5% (95%CI 97.1–99.9), 99.3% (95%CI 95.9–99.9), 92.9% (95%CI 68.5–98.7), and 86.7% (95%CI 62.1–96.3) through 6, 9, 12, or 24 months, respectively.

We also performed sensitivity analyses to examine signature performance among incident pulmonary TB cases only (**Figure S3D**; n=18, excluding 6 extra-pulmonary TB cases) and microbiologically-confirmed incident TB cases only (**Figure S3E**; n=12, excluding 12 clinically-diagnosed TB cases). There was no discernible effect on AUC when extra-pulmonary TB cases were excluded. There was only one microbiologically-confirmed incident TB case within 6 months; wide-confidence intervals precluded interpretation of the effect of microbiological versus clinical endpoints on prognostic performance.

Participant sex and HIV status, but not IPT, was associated with transcriptomic signature scores among non-progressors

In the multivariable linear regression analysis, female sex (**Figure S4**) and HIV status (both undetectable and detectable HIV viral loads; **Figure S5**) were most frequently associated with higher signature scores among non-progressors at enrolment (**Table S4A**) and month 6 of follow-up (**Table S4B**). Study site, age, BMI, tobacco smoking, and drinking had a negligible effect on some signature scores (**Table S4A-B**; other signature data not shown). As compared to not taking IPT, completion of 6 months of IPT had no discernible effect on signature scores among non-progressors (**Table S4A-B** and **Figure S6-S8**). Quantitative IGRA responses were not correlated with signature scores at enrolment (**Figure S9**). However, conversion from IGRA negative at enrolment to IGRA positive at month 6 was associated with significantly higher scores for four signatures (Francisco2, Gjoen7, Roe3, and Satproedprai7) at month 6 (data not shown). Duffy9 signature scores were among the least affected by confounding variables at both enrolment and month 6 measurement timepoints, and did not appear to be affected by HIV.

Supplementary Discussion

In the multivariable regression analysis, HIV status was associated with higher signature scores among non-progressors, likely due to activation of the same ISG pathways, as previously reported.[23,27-29] Signature scores were also higher among female participants, as previously seen.[29,30] Female sex has also been associated with moderately increased risk of IGRA-positivity among close contacts in Brazil.[31] The Duffy9 signature, which was trained as a multinomial signature to classify TB separately from HIV,[3] was among the least affected by confounding variables at both enrolment and month 6 measurement timepoints, and did not appear to be affected by HIV. The fewer sources of variability from confounding variables may partly explain the preservation of Duffy9 prognostic performance from 9 through 24 months follow-up.

Supplementary Tables

Table S1. TaqMan PCR primer-probe panel for Fluidigm 96.96 gene expression integrated fluidic circuit.

Gene Symbol	TaqMan Assay ID	Assay excluded**	Reference probe	# of signatures	da Costa3	de Araujo1	Duffy9 (10)†	Francisco2	Gjøen7	Gliddon3	Gliddon4	Jacobsen3	Kaforou22 (27)‡	Maertzdorf4	Penn-Nicholson6	Rajan5	Roe1	Roe3	Sambarey10	Satproedprai7	Suliman2	Suliman4	Sweeney3	Thompson5
ACTR3	Hs01029159_g1		X	14	X	X	X	X	X	X	X	X	X			X	X	X	X	X				
TMBIM6	Hs00162661_m1		X	14	X	X	X	X	X	X	X	X	X			X	X	X	X	X				
USF2	Hs01100994_g1		X	14	X	X	X	X	X	X	X	X	X			X	X	X	X	X				
ACTA2	Hs00426835_g1			1												X								
ANKRD22	Hs00944015_m1			2									X								X			
APOL1	Hs01066280_m1			1																X				
ARG1	Hs00968979_m1			1							X													
BATF2	Hs00912737_m1			2													X	X						
BCL6	AREPURD*			1															X					
BLK	Hs01017452_m1			1																		X		
C1QA	Hs00706358_s1	X		0																				
C1QB	Hs00608019_m1			3			X			X			‡											
C1QC	Hs00757779_m1	X		1									‡											
C4ORF18 / FAM198B	Hs00259260_s1			1									X											
C5	Hs00156197_m1			1									X											
CCR6	Hs01890706_s1			1									X											
CD160	Hs00199894_m1			1			X																	
CD1C	Hs00957534_g1			1																		X		
CD36.2	Hs01567186_m1			1			X																	
CD3E	APDJ3NT*			1					X															
CD40L	Hs00163934_m1			1			X																	
CD74-j1	Hs04983808_s1	X		0																				
CD74-j2	AP9HN47*	X		0																				
CD79A	Hs00998120_g1			1									X											
CD79B	Hs01058826_g1			1									X											
CDCA7	Hs00230589_m1	X		0																				
CDKN1C	Hs00175938_m1	X		0																				
CXCR5	Hs00540548_s1			1									X											
CYP4F3	Hs01587860_mH			1															X					
DUSP3	Hs01115776_m1			2									X										X	
F2RL1	Hs00608346_m1	X		0																				
FAM20A	Hs01034066_m1			1									X											
FCGR1A	Hs00174081_m1			5	X					X		X	X						X					
FCGR1A	AP2XDMK*			1																X				
FCGR1B	Hs02341825_m1			3			X						X		X									
FCGR1B_ VARIANT1	AP3267H*			1																X				
FCGR1B_ VARIANT2	AP47ZTF*			1																X				
FCGR1C	Hs00417598_m1			1									X											
FLVCR2	Hs00900390_m1			1									X											
GAS6	AR47XGZ*			2									X									X		
GAS6	AR323W3*			1									X											
GBP1	Hs00977005_m1			1										X										
GBP2	Hs00894846_g1			1											X									
GBP5	Hs00369472_m1			5	X			X	X									X					X	
GBP6	Hs01584201_m1			4			X				X		‡			X								
GNG7	Hs00192999_m1			1									X											
GYG1	Hs00907542_g1			1												X								
GZMA	Hs00989184_m1			1	X																			
HK3	Hs01092850_m1			1															X					

Gene Symbol	TaqMan Assay ID	Assay excluded**	Reference probe	# of signatures	da Costa3	de Araujo1	Duffy9 (10)†	Francisco2	Gjøen7	Gliddon3	Gliddon4	Jacobsen3	Kaforou22 (27)‡	Maertzdorf4	Penn-Nicholson6	Rajan5	Roe1	Roe3	Sambarey10	Satproedprai7	Suliman2	Suliman4	Sweeney3	Thompson5
ID3	Hs00954037_g1			2			X							X										
IFI44L	Hs00915292_m1			1															X					
IFITM3	Hs03057129_s1			2					X					X										
IFITM3_G	APMF24C*	X		0																				
KAZN	AP7DVDD*			1																X				
KIF1B	Hs01114512_g1			1					X															
KLF2	Hs00360439_g1			2				X															X	
KLF2	ARH6CF4*	X		0																				
KLHDC8B	Hs00293902_m1	X		0																				
LAG3	Hs00958444_g1			1			X																	
LHFPL2	Hs00299613_m1			1									X											
LTF	Hs00158924_m1			1								X												
MAFB	APZTH2N*			1																X				
MAP7D3	Hs00226257_m1			1																				X
MMP9	APEPW9P*			1					X															
MPO	Hs00165162_m1			1									X											
MTRF1L	Hs01097882_g1			1												X								
NOD2	Hs01550759_g1			1					X															
NPC2	Hs01119244_m1			1		X																		
OSBPL10	Hs00215016_m1			1																	X			
P2RY14	Hs01848195_s1			1										X										
PRDM1	Hs00153357_m1			1							X													
RAB13	Hs04400188_g1			1															X					
Rab33A	Hs00191243_m1			1								X												
RABL2A	Hs00255244_m1			1												X								
RBBP8	Hs01090329_m1			1															X					
RP11-295G20.2	Hs01373568_m1			1																				X
S100A8	Hs00374264_g1			1									X											
SCARF1	Hs01092483_m1			1														X						
SDR39U1	Hs01016970_g1			1											X									
SEPT4	Hs00910208_g1			1																		X		
SERPING1	Hs00934329_m1			1											X									
SLPI	Hs00268206_m1			1															X					
SMARCD3	Hs01088251_g1			3									X						X					X
STAT1	Hs01013996_m1			1																X				
STT3A	Hs00967491_m1			1																				X
TIMM10	APT2DEC*			1															X					
TMCC1	Hs01037666_s1			1							X													
TNIP1	AP47ZYZ*			1					X															
TRMT2A	Hs01000041_g1			1											X									
TUBGCP6	Hs00363509_g1			1											X									
UCP2	Hs01075224_g1			1																				X
VAMP5	Hs01105383_g1			1									X											
WARS-j1	Hs00998737_m1	X		0																				
WARS-j2	APAAFHX*	X		0																				
ZDHHC19	Hs00376118_m1			1			X																	
ZNF296	Hs00377132_m1			2						X			X											

Signatures are named by first author and number of transcripts included in the model (e.g. Author11). Numbers in brackets indicate the original number of transcripts in the published model. Some signatures have a reduced number of transcripts when translated to RT-qPCR due to duplicate transcript symbols (IDs), high-primer probe failure rate (**poor amplification efficiency), or where transcript sequences from an original discovery cohorts could not be mapped to a more recent reference transcriptome.

* Custom designed primer-probe assays; available on request to corresponding author.

† The CERKL1 primer-probe assay failed during panel optimisation and was removed from the Duffy10 signature.

‡ Three transcripts (C1QB, C1QC, and GBP6) were excluded from the Kaforou27 signature due to a high primer-probe assay failure rate (**). Unique primer-probe assays could not be designed for both of the FCGR1B transcript variants; only one FCGR1B primer-probe was included in the Kaforou22 model. We were unable to design a primer-probe for LOC728744; all information had been withdrawn from both the NCBI and RefSeq databases.

Table S2. Characteristics of the close-contact study population with transcriptomic signature scores.

Characteristic	Non-progressors, N = 1,765	Progressors, N = 24	p-value [a]
Female, n (%)	1,045 (59.2)	16 (66.7)	0.46
Median age, years (IQR)	32.0 (16.0-46.5)	29.3 (18.3-49.1)	0.54
City, n (%)			**0.001**
Manaus	824 (46.8)	3 (12.5)	
Rio de Janeiro	649 (36.9)	14 (58.3)	
Salvador	287 (16.3)	7 (29.2)	
Missing	5	0	
Ethnicity, n (%)			0.46
Pardo	1,050 (59.5)	11 (45.8)	
Black	355 (20.1)	7 (29.2)	
White	341 (19.3)	6 (25.0)	
Indian	13 (0.7)	0 (0.0)	
Asian	5 (0.3)	0 (0.0)	
Missing	1	0	
Smoking history, n (%)			0.65
Never	1,299 (73.6)	20 (83.3)	
Former	287 (16.3)	2 (8.3)	
Current	178 (10.1)	2 (8.3)	
Missing	1	0	
Alcohol consumption history, n (%)			0.15
Never	821 (46.5)	16 (66.7)	
Former	340 (19.3)	2 (8.3)	
Current	603 (34.2)	6 (25.0)	
Missing	1	0	
Median body-mass index (BMI), kg/m^2 (IQR)	24.4 (20.2-28.7)	23.8 (19.4-26.2)	0.41
Missing	1	0	
HIV positive, n (%)	46 (2.6)	0 (0.0)	NA
HIV plasma viral load, n (%)			NA
Undetectable (<50 copies/mL)	14 (48.3)	0 (0.0)	
Detectable (>50 copies/mL)	15 (51.7)	0 (0.0)	
Missing	17	0	
Previous TB, n (%)	73 (4.2)	4 (16.7)	**0.018**
Missing	6	0	
Baseline IGRA result, n (%)			**<0.001**
Negative	1,088 (61.8)	5 (20.8)	
Positive	642 (36.5)	19 (79.2)	
Indeterminate	31 (1.8)	0 (0.0)	
Missing	4	0	
Median baseline IGRA response, IU/mL (IQR)	0.1 (0.0-1.2)	1.9 (0.4-5.9)	**<0.001**
Missing	35	0	
Month 6 IGRA status, [b] n (%)			**<0.001**
Persistent negative	787 (51.1)	4 (16.7)	
Converter (Baseline negative)	112 (7.3)	1 (4.2)	
Baseline positive	642 (41.7)	19 (79.2)	
Missing	224	0	
Isoniazid preventive therapy (IPT), n (%)			0.54
On IPT at enrolment	10 (0.6)	0 (0.0)	
Started IPT during study	439 (24.9)	4 (16.7)	
No IPT received [c]	1,316 (74.6)	20 (83.3)	
Completed IPT during study, n/N (%)			0.051
Completed 168 (6x28) days	313/449 (69.7)	1/4 (25.0)	
Completed < 168 (6x28) days	113/449 (25.2)	2/4 (50.0)	
No IPT end date [d]	23/449 (5.1)	1/4 (25.0)	
No IPT received [c]	1,316	20	
Median IPT received during study, [e] days (IQR)	188.0 (160.2-250.8)	48.0 (34.5-173.0)	0.33
Completed IPT by month 6, n/N (%)			0.17
Completed 168 (6x28) days	206/449 (45.9)	1/4 (25.0)	
Completed < 168 (6x28) days	69/449 (15.5)	0/4 (0.0)	
Started IPT after 2nd PAXgene draw	51/449 (11.4)	0/4 (0.0)	
No repeat PAXgene collected	100/449 (22.3)	2/4 (50.0)	
No IPT end date [d]	23/449 (5.1)	1/4 (25.0)	
No IPT received [c]	1,316	20	

[a] Pearson's Chi-squared test; Wilcoxon rank sum test; Fisher's exact test.

[b] Repeat interferon-γ release assay (IGRA) testing was performed at month 6 of follow-up among baseline IGRA negative (< 0.35 IU/mL) participants only. Participants with baseline and month 6 negative IGRA results are termed "persistent negative". "Converters" are those participants whose IGRA are negative at baseline, but convert to positive (≥ 0.35 IU/mL) at the month 6 visit.

[c] No isoniazid preventive therapy (IPT) recorded as having been received during the study.

[d] Started isoniazid preventive therapy (IPT) during study, but no treatment end date recorded during the study.

[e] Only includes participants with recorded IPT start and end dates.

Table S3A. Transcriptomic signature prognostic sensitivity and specificity benchmarked against the minimum WHO TPP for test for progression to TB disease, through 6, 9, 12, and 24 months of follow-up. All study participants with transcriptomic signature scores at enrolment included.

	Accuracy through 6 months follow-up		Accuracy through 9 months follow-up		Accuracy through 12 months follow-up		Accuracy through 24 months follow-up	
**Signature**	**Sensitivity, % (95% CI)**	**Specificity, % (95% CI)**	**Sensitivity, % (95% CI)**	**Specificity, % (95% CI)**	**Sensitivity, % (95% CI)**	**Specificity, % (95% CI)**	**Sensitivity, % (95% CI)**	**Specificity, % (95% CI)**
QuantiFERON TB-Gold (≥0.35 IU/mL)[a]	100 (67.6-100)	62.8 (60.5-65.0)	90.9 (62.3- 98.4)	62.8 (60.5-65.0)	86.7 (62.1-96.3)	62.8 (60.5-65.0)	79.2 (59.5-90.8)	62.8 (60.5-65.0)
QuantiFERON TB-Gold (sensitivity ≥75%)[b]	75.0 (40.9-92.9)	79.7 (77.7-81.5)	81.8 (52.3- 94.9)	65.5 (63.3-67.8)	80.0 (54.8-93.0)	65.5 (63.2-67.7)	75.0 (55.1-88.0)	65.5 (63.2-67.7)
Gliddon4	85.7 (48.7-97.4)	86.3 (84.5-88.0)	77.8 (45.3- 93.7)	86.3 (84.5-88.0)	81.8 (52.3-94.9)	65.8 (63.3-68.1)	78.9 (56.7-91.5)	28.8 (26.5-31.1)
Kaforou22	85.7 (48.7-97.4)	70.5 (68.0-72.9)	77.8 (45.3- 93.7)	70.5 (68.0-72.9)	75.0 (46.8-91.1)	43.2 (40.5-45.9)	78.9 (56.7-91.5)	35.4 (32.9-38.0)
Satproedprai7	75.0 (40.9-92.9)	66.5 (64.2-68.7)	81.8 (52.3- 94.9)	56.3 (53.9-58.6)	80.0 (54.8-93.0)	56.3 (53.9-58.6)	75.0 (55.1-88.0)	56.3 (53.9-58.6)
Suliman4	75.0 (40.9-92.9)	81.3 (79.4-83.1)	81.8 (52.3- 94.9)	48.4 (46.0-50.7)	80.0 (54.8-93.0)	48.4 (46.0-50.7)	75.0 (55.1-88.0)	46.3 (44.0-48.6)
Rajan5	85.7 (48.7-97.4)	74.7 (72.5-76.8)	77.8 (45.3- 93.7)	74.7 (72.5-76.8)	81.8 (52.3-94.9)	56.1 (53.7-58.6)	77.8 (54.8-91.0)	54.3 (51.8-56.8)
Roe1	75.0 (40.9-92.9)	78.8 (76.9-80.7)	80.0 (49.0- 94.3)	73.1 (71.0-75.1)	78.6 (52.4-92.4)	54.3 (52.0-56.6)	78.3 (58.1-90.3)	45.0 (42.6-47.3)
Penn-Nicholson6	75.0 (40.9-92.9)	75.4 (73.3-77.3)	81.8 (52.3- 94.9)	28.2 (26.1-30.3)	80.0 (54.8-93.0)	19.3 (17.5-21.2)	75.0 (55.1-88.0)	28.2 (26.1-30.3)
Duffy9	85.7 (48.7-97.4)	71.4 (69.0-73.7)	77.8 (45.3- 93.7)	74.6 (72.2-76.8)	81.8 (52.3-94.9)	73.6 (71.2-75.8)	77.8 (54.8-91.0)	55.4 (52.7-57.9)
Jacobsen3	75.0 (40.9-92.9)	69.3 (67.1-71.4)	81.8 (52.3- 94.9)	56.3 (54.0-58.6)	80.0 (54.8-93.0)	36.0 (33.8-38.3)	75.0 (55.1-88.0)	38.7 (36.5-41.0)
Gliddon3	85.7 (48.7-97.4)	46.9 (44.4-49.3)	80.0 (49.0- 94.3)	46.9 (44.4-49.3)	78.6 (52.4-92.4)	58.2 (55.8-60.6)	77.3 (56.6-89.9)	56.2 (53.8-58.6)
Gjoen7	75.0 (40.9-92.9)	74.0 (71.9-76.0)	81.8 (52.3- 94.9)	67.3 (65.0-69.4)	80.0 (54.8-93.0)	46.5 (44.1-48.8)	75.0 (55.1-88.0)	35.3 (33.1-37.6)
Roe3	75.0 (40.9-92.9)	79.1 (77.1-81.0)	80.0 (49.0- 94.3)	64.2 (61.9-66.4)	78.6 (52.4-92.4)	57.6 (55.3-59.9)	78.3 (58.1-90.3)	44.3 (42.0-46.7)
Sambarey10	75.0 (40.9-92.9)	65.7 (63.5-67.9)	81.8 (52.3- 94.9)	49.2 (46.9-51.6)	80.0 (54.8-93.0)	49.2 (46.9-51.6)	75.0 (55.1-88.0)	49.2 (46.9-51.6)
Suliman2	75.0 (40.9-92.9)	71.9 (69.8-74.0)	81.8 (52.3- 94.9)	25.4 (23.4-27.5)	80.0 (54.8-93.0)	25.4 (23.4-27.5)	75.0 (55.1-88.0)	32.9 (30.7-35.1)
Sweeney3	75.0 (40.9-92.9)	72.3 (70.1-74.3)	81.8 (52.3- 94.9)	29.5 (27.4-31.7)	80.0 (54.8-93.0)	27.7 (25.6-29.8)	75.0 (55.1-88.0)	31.0 (28.9-33.2)
Maertzdorf4	75.0 (40.9-92.9)	48.6 (46.2-50.9)	81.8 (52.3- 94.9)	42.8 (40.5-45.2)	80.0 (54.8-93.0)	7.3 (6.1- 8.6)	75.0 (55.1-88.0)	42.8 (40.5-45.2)
Francisco2	75.0 (40.9-92.9)	75.3 (73.2-77.2)	81.8 (52.3- 94.9)	33.7 (31.5-36.0)	80.0 (54.8-93.0)	33.7 (31.5-36.0)	75.0 (55.1-88.0)	33.7 (31.5-36.0)
Thompson5	75.0 (40.9-92.9)	50.2 (47.9-52.6)	81.8 (52.3- 94.9)	20.4 (18.5-22.3)	80.0 (54.8-93.0)	24.0 (22.1-26.1)	75.0 (55.1-88.0)	24.0 (22.1-26.1)
de Araujo1	75.0 (40.9-92.9)	51.1 (48.7-53.4)	81.8 (52.3- 94.9)	9.8 (8.5-11.3)	80.0 (54.8-93.0)	12.7 (11.2-14.4)	75.0 (55.1-88.0)	13.0 (11.5-14.7)
da Costa3	100 (67.6-100)	0.0 (0.0-0.2)	100 (74.1-100)	0.0 (0.0- 0.2)	100 (79.6-100)	0.0 (0.0-0.2)	75.0 (55.1-88.0)	31.7 (29.5-33.9)

Transcriptomic signatures are sorted by prognostic performance (area under the curve, AUC) through 6 months of follow-up. Specificity is reported at a sensitivity ≥75% for each signature, at each timepoint, unless otherwise stated.
[a] Sensitivity and specificity reported at QuantiFERON TB-Gold manufacturer positivity cut-off (≥0.35 IU/mL).
[b] QuantiFERON TB-Gold specificity reported at a sensitivity ≥75%.

Table S3B. Sensitivity analysis among study participants who did not receive IPT during the study: Transcriptomic signature prognostic performance.

	Healthy non-progressors	TB cases through 6 months	TB cases through 9 months	TB cases through 12 months	TB cases through 24 months	AUC through 6 months follow-up (95% CI)	AUC through 9 months follow-up (95% CI)	AUC through 12 months follow-up (95% CI)	AUC through 24 months follow-up (95% CI)
**QuantiFERON TB-Gold***	1283	6	9	13	20	0.92 (0.87-0.97)	0.85 (0.68-1)	0.82 (0.70-0.95)	0.79 (0.68-0.90)
**Gliddon4**	1123	5	7	9	16	0.88 (0.77-0.99)	0.78 (0.54-1)	0.70 (0.46-0.94)	0.65 (0.47-0.83)
**Rajan5**	1129	5	7	9	15	0.87 (0.79-0.94)	0.84 (0.73-0.94)	0.76 (0.64-0.89)	0.66 (0.53-0.79)
**Kaforou22**	990	5	7	10	16	0.82 (0.67-0.98)	0.76 (0.56-0.97)	0.64 (0.45-0.83)	0.63 (0.49-0.77)
**Satproedprai7**	1245	6	9	13	20	0.79 (0.64-0.94)	0.72 (0.55-0.88)	0.67 (0.54-0.80)	0.65 (0.53-0.76)
**Roe1**	1301	6	8	12	19	0.78 (0.63-0.94)	0.72 (0.54-0.91)	0.70 (0.57-0.82)	0.62 (0.49-0.74)
**Suliman4**	1313	6	9	13	20	0.77 (0.58-0.97)	0.71 (0.49-0.92)	0.69 (0.53-0.85)	0.67 (0.55-0.79)
**Duffy9**	1032	5	7	9	15	0.76 (0.49-1)	0.82 (0.62-1)	0.80 (0.64-0.96)	0.69 (0.57-0.82)
**Roe3**	1301	6	8	12	19	0.75 (0.50-0.99)	0.72 (0.51-0.94)	0.69 (0.55-0.84)	0.64 (0.51-0.76)
**Penn-Nicholson6**	1312	6	9	13	20	0.74 (0.54-0.94)	0.64 (0.42-0.85)	0.55 (0.37-0.72)	0.57 (0.42-0.71)
**Jacobsen3**	1304	6	9	13	20	0.74 (0.53-0.96)	0.71 (0.53-0.89)	0.64 (0.48-0.79)	0.61 (0.48-0.74)
**Sambarey10**	1304	6	9	13	20	0.74 (0.52-0.96)	0.71 (0.54-0.89)	0.67 (0.52-0.81)	0.64 (0.52-0.76)
**Gjoen7**	1316	6	9	13	20	0.74 (0.51-0.97)	0.69 (0.51-0.88)	0.65 (0.51-0.79)	0.58 (0.44-0.71)
**Gliddon3**	1195	5	8	12	18	0.73 (0.05-0.97)	0.70 (0.51-0.89)	0.69 (0.56-0.81)	0.68 (0.58-0.78)
**Suliman2**	1306	6	9	13	20	0.7 (0.45-0.94)	0.61 (0.37-0.85)	0.59 (0.40-0.78)	0.61 (0.47-0.75)
**Sweeney3**	1313	6	9	13	20	0.67 (0.36-0.98)	0.67 (0.43-0.90)	0.60 (0.41-0.78)	0.58 (0.44-0.71)
**Maertzdorf4**	1274	6	9	13	20	0.66 (0.46-0.86)	0.42 (0.21-0.63)	0.44 (0.29-0.60)	0.42 (0.30-0.54)
**Francisco2**	1313	6	9	13	20	0.65 (0.33-0.98)	0.65 (0.41-0.89)	0.60 (0.42-0.78)	0.57 (0.43-0.71)
**da Costa3**	1316	6	9	13	20	0.60 (0.40-0.80)	0.62 (0.46-0.78)	0.53 (0.38-0.68)	0.57 (0.45-0.70)
**Thompson5**	1315	6	9	13	20	0.60 (0.32-0.88)	0.46 (0.21-0.71)	0.50 (0.32-0.69)	0.46 (0.31-0.61)
**de Araujo1**	1313	6	9	13	20	0.57 (0.26-0.87)	0.50 (0.25-0.76)	0.51 (0.32-0.71)	0.58 (0.43-0.72)

Transcriptomic signatures are sorted by prognostic performance (area under the curve, AUC) through 6 months of follow-up.
* QuantiFERON TB-Gold AUCs were calculated using quantitative results.

Table S3C. Sensitivity analysis among study participants who did not receive IPT during the study: Transcriptomic signature prognostic sensitivity and specificity benchmarked against the minimum WHO TPP for test for progression to TB disease, through 6, 9, 12, and 24 months of follow-up. Only study participants with transcriptomic signature scores at enrolment who did not receive IPT during the study included.

	Accuracy through 6 months follow-up		Accuracy through 9 months follow-up		Accuracy through 12 months follow-up		Accuracy through 24 months follow-up	
**Signature**	**Sensitivity, % (95% CI)**	**Specificity, % (95% CI)**	**Sensitivity, % (95% CI)**	**Specificity, % (95% CI)**	**Sensitivity, % (95% CI)**	**Specificity, % (95% CI)**	**Sensitivity, % (95% CI)**	**Specificity, % (95% CI)**
QuantiFERON TB-Gold (≥0.35 IU/mL)[a]	100 (61.0-100)	79.4 (77.1-81.5)	88.9 (56.5-98.0)	79.4 (77.1-81.5)	84.6 (57.8-95.7)	79.4 (77.1-81.5)	75.0 (53.1-88.8)	79.4 (77.1-81.5)
QuantiFERON TB-Gold (sensitivity ≥75%)[b]	83.3 (43.6-97.0)	88.9 (87.0-90.5)	77.8 (45.3-93.7)	88.9 (87.0-90.5)	76.9 (49.7-91.8)	81.3 (79.1-83.3)	75.0 (53.1-88.8)	79.9 (77.6-82.0)
Gliddon4	80.0 (37.6-96.4)	90.0 (88.1-91.6)	85.7 (48.7-97.4)	66.1 (63.3-68.8)	77.8 (45.3-93.7)	66.1 (63.3-68.8)	75.0 (50.5-89.8)	28.3 (25.8-31.0)
Rajan5	80.0 (37.6-96.4)	82.5 (80.1-84.6)	85.7 (48.7-97.4)	75.8 (73.2-78.2)	77.8 (45.3-93.7)	57.6 (54.7-60.4)	80.0 (54.8-93.0)	43.2 (40.4-46.1)
Kaforou22	80.0 (37.6-96.4)	70.9 (68.0-73.7)	85.7 (48.7-97.4)	59.0 (55.9-62.0)	80.0 (49.0-94.3)	33.2 (30.4-36.2)	75.0 (50.5-89.8)	34.7 (31.8-37.8)
Satproedprai7	83.3 (43.6-97.0)	63.3 (60.6-65.9)	77.8 (45.3-93.7)	57.6 (54.8-60.3)	76.9 (49.7-91.8)	57.6 (54.8-60.3)	75.0 (53.1-88.8)	42.3 (39.6-45.1)
Roe1	83.3 (43.6-97.0)	73.3 (70.8-75.6)	75.0 (40.9-92.9)	73.3 (70.8-75.6)	75.0 (46.8-91.1)	54.6 (51.9-57.3)	78.9 (56.7-91.5)	38.8 (36.2-41.5)
Suliman4	83.3 (43.6-97.0)	47.8 (45.1-50.5)	77.8 (45.3-93.7)	47.8 (45.1-50.5)	76.9 (49.7-91.8)	47.8 (45.1-50.5)	75.0 (53.1-88.8)	47.8 (45.1-50.5)
Duffy9	80.0 (37.6-96.4)	71.4 (68.6-74.1)	85.7 (48.7-97.4)	71.4 (68.6-74.1)	77.8 (45.3-93.7)	73.7 (71.0-76.3)	80.0 (54.8-93.0)	53.1 (50.1-56.1)
Roe3	83.3 (43.6-97.0)	64.6 (62.0-67.2)	75.0 (40.9-92.9)	64.6 (62.0-67.2)	75.0 (46.8-91.1)	59.3 (56.6-62.0)	78.9 (56.7-91.5)	37.9 (35.3-40.6)
Penn-Nicholson6	83.3 (43.6-97.0)	70.7 (68.2-73.1)	77.8 (45.3-93.7)	29.0 (26.6-31.5)	76.9 (49.7-91.8)	19.3 (17.2-21.5)	75.0 (53.1-88.8)	25.9 (23.6-28.4)
Jacobsen3	83.3 (43.6-97.0)	66.0 (63.4-68.5)	77.8 (45.3-93.7)	57.9 (55.2-60.6)	76.9 (49.7-91.8)	37.5 (34.9-40.2)	75.0 (53.1-88.8)	37.5 (34.9-40.2)
Sambarey10	83.3 (43.6-97.0)	54.0 (51.3-56.7)	77.8 (45.3-93.7)	51.2 (48.4-53.9)	76.9 (49.7-91.8)	51.2 (48.4-53.9)	75.0 (53.1-88.8)	51.2 (48.4-53.9)
Gjoen7	83.3 (43.6-97.0)	69.4 (66.8-71.8)	77.8 (45.3-93.7)	68.5 (65.9-70.9)	76.9 (49.7-91.8)	48.7 (46.0-51.4)	75.0 (53.1-88.8)	28.0 (25.7-30.5)
Gliddon3	80.0 (37.6-96.4)	48.2 (45.4-51.0)	75.0 (40.9-92.9)	48.2 (45.4-51.0)	75.0 (46.8-91.1)	58.9 (56.1-61.7)	77.8 (54.8-91.0)	50.7 (47.9-53.5)
Suliman2	83.3 (43.6-97.0)	49.9 (47.2-52.6)	77.8 (45.3-93.7)	25.9 (23.6-28.3)	76.9 (49.7-91.8)	25.9 (23.6-28.3)	75.0 (53.1-88.8)	33.5 (31.0-36.1)
Sweeney3	83.3 (43.6-97.0)	30.1 (27.7-32.6)	77.8 (45.3-93.7)	30.1 (27.7-32.6)	76.9 (49.7-91.8)	28.2 (25.8-30.7)	75.0 (53.1-88.8)	30.1 (27.7-32.6)
Maertzdorf4	83.3 (43.6-97.0)	42.9 (40.2-45.6)	77.8 (45.3-93.7)	7.5 (6.1- 9.0)	76.9 (49.7-91.8)	20.3 (18.1-22.5)	75.0 (53.1-88.8)	20.3 (18.1-22.5)
Francisco2	83.3 (43.6-97.0)	26.0 (23.7-28.4)	77.8 (45.3-93.7)	35.1 (32.6-37.7)	76.9 (49.7-91.8)	35.1 (32.6-37.7)	75.0 (53.1-88.8)	30.2 (27.8-32.8)
da Costa3	83.3 (43.6-97.0)	57.1 (54.4-59.7)	77.8 (45.3-93.7)	57.1 (54.4-59.7)	76.9 (49.7-91.8)	34.6 (32.1-37.2)	75.0 (53.1-88.8)	34.6 (32.1-37.2)
Thompson5	83.3 (43.6-97.0)	20.0 (17.9-22.2)	77.8 (45.3-93.7)	8.5 (7.1-10.1)	76.9 (49.7-91.8)	15.8 (13.9-17.9)	75.0 (53.1-88.8)	15.7 (13.9-17.8)
de Araujo1	83.3 (43.6-97.0)	9.4 (7.9-11.1)	77.8 (45.3-93.7)	9.4 (7.9-11.1)	76.9 (49.7-91.8)	12.5 (10.8-14.4)	75.0 (53.1-88.8)	29.3 (26.9-31.8)

Transcriptomic signatures are sorted by prognostic performance (area under the curve, AUC) through 6 months of follow-up. Specificity is reported at a sensitivity ≥75% for each signature, at each timepoint, unless otherwise stated.
[a] Sensitivity and specificity reported at QuantiFERON TB-Gold manufacturer positivity cut-off (≥0.35 IU/mL).
[b] QuantiFERON TB-Gold specificity reported at a sensitivity ≥75%.

Table S3D. Sensitivity analysis among study participants who did not receive IPT during the study: Transcriptomic signature prognostic positive and negative predictive values benchmarked against the minimum WHO TPP for test for progression to TB disease, through 6, 9, 12, and 24 months of follow-up. Only study participants with transcriptomic signature scores at enrolment who did not receive IPT during the study included.

	Accuracy through 6 months follow-up		Accuracy through 9 months follow-up		Accuracy through 12 months follow-up		Accuracy through 24 months follow-up	
**Signature**	**NPV, % (95% CI)**	**PPV, % (95% CI)**	**NPV, % (95% CI)**	**PPV, % (95% CI)**	**NPV, % (95% CI)**	**PPV, % (95% CI)**	**NPV, % (95% CI)**	**PPV, % (95% CI)**
QuantiFERON TB-Gold (≥0.35 IU/mL)[a]	100 (99.6-100)	2.2 (1.0-4.8)	99.9 (99.4-100)	2.9 (1.5-5.7)	99.8 (99.3-99.9)	4.0 (2.2-7.0)	99.5 (98.9- 99.8)	5.4 (3.3-8.7)
QuantiFERON TB-Gold (sensitivity ≥75%)[b]	99.9 (99.5-100)	3.4 (1.5-7.7)	99.8 (99.4-100)	4.7 (2.3-9.3)	99.7 (99.2-99.9)	4.0 (2.2-7.2)	99.5 (98.9-99.8)	5.5 (3.4-8.9)
Gliddon4	99.9 (99.4-100)	3.4 (1.3-8.5)	99.9 (99.2-100)	1.6 (0.7-3.3)	99.7 (99.0-99.9)	1.8 (0.9-3.7)	98.8 (96.8-99.5)	1.5 (0.8-2.5)
Rajan5	99.9 (99.4-100)	2.0 (0.8-5.0)	99.9 (99.3-100)	2.2 (1.0-4.6)	99.7 (98.9-99.9)	1.4 (0.7-2.9)	99.4 (98.2-99.8)	1.8 (1.1-3.2)
Kaforou22	99.9 (99.2-100)	1.4 (0.5-3.5)	99.8 (99.0-100)	1.5 (0.7-3.1)	99.4 (97.8-99.8)	1.2 (0.6-2.3)	98.9 (97.1-99.6)	1.8 (1.0-3.2)
Satproedprai7	99.9 (99.3-100)	1.1 (0.5-2.5)	99.7 (99.0-99.9)	1.3 (0.6-2.7)	99.6 (98.8-99.9)	1.9 (1.0-3.4)	99.1 (97.8-99.6)	2.0 (1.2-3.3)
Roe1	99.9 (99.4-100)	1.4 (0.6-3.3)	99.8 (99.2-99.9)	1.7 (0.8-3.6)	99.6 (98.8-99.9)	1.5 (0.8-2.8)	99.2 (98.0-99.7)	1.8 (1.1-3.0)
Suliman4	99.8 (99.1-100)	0.7 (0.3-1.7)	99.7 (98.8-99.9)	1.0 (0.5-2.1)	99.5 (98.6-99.8)	1.4 (0.8-2.6)	99.2 (98.2-99.7)	2.1 (1.3-3.5)
Duffy9	99.9 (99.2-100)	1.3 (0.5-3.4)	99.9 (99.2-100)	2.0 (0.9-4.3)	99.7 (99.0-99.9)	2.5 (1.2-5.1)	99.5 (98.4-99.8)	2.4 (1.4-4.2)
Roe3	99.9 (99.3-100)	1.1 (0.5-2.5)	99.8 (99.1-99.9)	1.3 (0.6-2.8)	99.6 (98.9-99.9)	1.7 (0.9-3.1)	99.2 (97.9-99.7)	1.8 (1.1-3.0)
Penn-Nicholson6	99.9 (99.4-100)	1.3 (0.6-3.0)	99.5 (98.1-99.9)	0.7 (0.4-1.5)	98.8 (96.6-99.6)	0.9 (0.5-1.7)	98.6 (96.7-99.4)	1.5 (0.9-2.5)
Jacobsen3	99.9 (99.3-100)	1.1 (0.5-2.6)	99.7 (99.0-99.9)	1.3 (0.6-2.6)	99.4 (98.2-99.8)	1.2 (0.7-2.2)	99.0 (97.7-99.6)	1.8 (1.1-3.0)
Sambarey10	99.9 (99.2-100)	0.8 (0.4-1.9)	99.7 (98.9-99.9)	1.1 (0.5-2.2)	99.6 (98.7-99.8)	1.5 (0.8-2.8)	99.3 (98.3-99.7)	2.3 (1.4-3.8)
Gjoen7	99.9 (99.4-100)	1.2 (0.5-2.8)	99.8 (99.2-99.9)	1.7 (0.8-3.4)	99.5 (98.6-99.8)	1.5 (0.8-2.7)	98.7 (96.9-99.4)	1.6 (0.9-2.6)
Gliddon3	99.8 (99.0-100)	0.6 (0.2-1.6)	99.7 (98.7-99.9)	1.0 (0.4-2.1)	99.6 (98.8-99.9)	1.8 (0.9-3.4)	99.3 (98.3-99.7)	2.3 (1.4-3.9)
Suliman2	99.8 (99.1-100)	0.8 (0.3-1.8)	99.4 (97.9-99.8)	0.7 (0.3-1.5)	99.1 (97.4-99.7)	1.0 (0.6-1.9)	98.9 (97.4-99.5)	1.7 (1.0-2.8)
Sweeney3	99.7 (98.6-100)	0.5 (0.2-1.3)	99.5 (98.2-99.9)	0.8 (0.4-1.6)	99.2 (97.7-99.7)	1.0 (0.6-1.9)	98.8 (97.1-99.5)	1.6 (1.0-2.6)
Maertzdorf4	99.8 (99.0-100)	0.7 (0.3-1.6)	97.9 (92.8-99.4)	0.6 (0.3-1.2)	98.9 (96.7-99.6)	1.0 (0.5-1.8)	98.1 (95.6-99.2)	1.5 (0.9-2.4)
Francisco2	99.7 (98.4-99.9)	0.5 (0.2-1.2)	99.6 (98.4-99.9)	0.8 (0.4-1.7)	99.4 (98.1-99.8)	1.2 (0.6-2.1)	98.8 (97.1-99.5)	1.6 (1.0-2.6)
da Costa3	99.9 (99.3-100)	0.9 (0.4-2.0)	99.7 (99.0-99.9)	1.2 (0.6-2.5)	99.3 (98.1-99.8)	1.1 (0.6-2.1)	98.9 (97.5-99.5)	1.7 (1.0-2.8)
Thompson5	99.6 (97.9-99.9)	0.5 (0.2-1.1)	98.2 (93.8-99.5)	0.6 (0.3-1.2)	98.6 (95.9-99.5)	0.9 (0.5-1.6)	97.6 (94.6-99.0)	1.3 (0.8-2.2)
de Araujo1	99.2 (95.6-99.9)	0.4 (0.2-1.0)	98.4 (94.4-99.6)	0.6 (0.3-1.2)	98.2 (94.9-99.4)	0.9 (0.5-1.6)	98.7 (97.0-99.5)	1.6 (1.0-2.6)

Transcriptomic signatures are sorted by prognostic performance (area under the curve, AUC) through 6 months of follow-up. Positive predictive value (PPV) and negative predictive value (NPV) are reported at a sensitivity ≥75% for each signature, at each timepoint, unless otherwise stated. See **Table S3C**.
[a] NPV and PPV reported at QuantiFERON TB-Gold manufacturer positivity cut-off (≥0.35 IU/mL).
[b] QuantiFERON TB-Gold NPV and PPV reported at a sensitivity ≥75%.

Table S4A. Multivariable linear regression analysis examining the effect of clinical variables on Gliddon4, Kaforou22, and Duffy9 scores among non-progressors at baseline.

	**Gliddon4**		**Kaforou22**		**Duffy9**	
	**β (95% CI)**	**p-value**	**β (95% CI)**	**p-value**	**β (95% CI)**	**p-value [a]**
(Intercept)	0.47 (0.32; 0.62)	<0.001	0.32 (0.14; 0.5)	0.005	0.56 (0.34; 0.77)	<0.001
Sex						
Male	Reference		Reference		Reference	
Female	0.06 (0.04; 0.08)	**<0.001**	0 (-0.02; 0.03)	0.93	0.02 (-0.01; 0.06)	0.54
Age (per 10 years)	0 (0; 0.01)	0.64	0.02 (0.01; 0.03)	**0.002**	0.01 (0; 0.02)	0.22
City						
Manaus	Reference		Reference		Reference	
Rio de Janeiro	0.03 (0.01; 0.06)	0.072	0.01 (-0.02; 0.04)	0.75	0.01 (-0.03; 0.05)	0.89
Salvador	0.01 (-0.02; 0.04)	0.82	-0.07 (-0.11; -0.03)	**0.007**	0.04 (-0.01; 0.09)	0.42
Ethnicity						
Pardo	Reference		Reference		Reference	
Black	-0.02 (-0.05; 0.01)	0.40	0 (-0.03; 0.04)	0.93	0 (-0.04; 0.04)	>0.99
White	-0.04 (-0.07; -0.01)	0.078	-0.03 (-0.07; 0.01)	0.31	-0.02 (-0.07; 0.02)	0.59
Indian	0.01 (-0.12; 0.13)	0.97	-0.07 (-0.22; 0.08)	0.66	-0.24 (-0.44; -0.05)	0.074
Asian	-0.05 (-0.27; 0.16)	0.84	0.07 (-0.18; 0.31)	0.82	0.01 (-0.3; 0.32)	0.98
Smoking history						
Never smoked	Reference		Reference		Reference	
Former smoker	0 (-0.04; 0.03)	0.98	0 (-0.04; 0.04)	0.98	0 (-0.05; 0.05)	>0.99
Current smoker	-0.01 (-0.05; 0.03)	0.90	0.04 (-0.01; 0.09)	0.35	0 (-0.06; 0.06)	0.97
Drinking history						
Never drunk	Reference		Reference		Reference	
Former drinker	0.01 (-0.03; 0.04)	0.89	-0.01 (-0.05; 0.03)	0.89	0.01 (-0.04; 0.06)	0.91
Current drinker	-0.01 (-0.03; 0.02)	0.89	0 (-0.03; 0.04)	>0.99	0 (-0.05; 0.04)	0.97
Body-mass index (per 10 kg/m^2)	0.02 (0; 0.04)	0.11	0.05 (0.03; 0.08)	**<0.001**	-0.03 (-0.06; 0)	0.15
HIV status						
HIV negative	Reference		Reference		Reference	
HIV+ VL undetectable [b]	0.16 (0.04; 0.28)	0.056	0.16 (0.01; 0.3)	0.15	0.03 (-0.15; 0.22)	0.90
HIV+ VL detectable [b]	0.16 (0.04; 0.28)	0.054	0.27 (0.13; 0.42)	**0.002**	0.15 (-0.04; 0.33)	0.34
Previous TB						
No	Reference		Reference		Reference	
Yes	-0.03 (-0.09; 0.02)	0.54	-0.07 (-0.14; -0.01)	0.15	-0.01 (-0.09; 0.08)	0.96
Isoniazid preventive therapy						
On IPT at enrolment	Reference		Reference		Reference	
Started IPT during study	-0.09 (-0.23; 0.06)	0.54	-0.04 (-0.22; 0.14)	0.87	-0.05 (-0.26; 0.16)	0.84
No IPT recorded [c]	-0.05 (-0.2; 0.09)	0.74	-0.01 (-0.18; 0.17)	0.98	0.01 (-0.2; 0.22)	0.98
Baseline IGRA result						
Negative	Reference		Reference		Reference	
Positive	0.01 (-0.02; 0.04)	0.72	0.01 (-0.03; 0.05)	0.85	0.03 (-0.02; 0.07)	0.54
**Summary Statistics**						
Adjusted R^2	0.05		0.09		0.01	
Missing variables	246		383		338	
Degrees of freedom	1159		1022		1067	

[a] Correction for false discovery was performed using the Benjamini and Hochberg method.[26]
[b] HIV plasma viral load (VL) < 50 copies/mL was considered undetectable, and ≥ 50 copies/mL was considered detectable.
[c] No isoniazid preventive therapy (IPT) recorded during study.

HIV+, HIV positive. VL, viral load. IPT, isoniazid preventive therapy. IGRA, interferon-γ release assay.

Table S4B. Multivariable linear regression analysis examining the effect of clinical variables on Gliddon4, Kaforou22, and Duffy9 scores among non-progressors at month 6 of follow-up.

	Gliddon4		Kaforou22		Duffy9	
	**β (95% CI)**	**p-value**	**β (95% CI)**	**p-value**	**β (95% CI)**	**p-value [a]**
(Intercept)	0.45 (0.39; 0.51)	<0.001	0.36 (0.28; 0.43)	<0.001	0.54 (0.45; 0.63)	<0.001
Sex						
Male	Reference		Reference		Reference	
Female	0.06 (0.03; 0.08)	**<0.001**	0.05 (0.02; 0.08)	**0.004**	0.01 (-0.03; 0.04)	0.91
Age (per 10 years)	0 (-0.01; 0.01)	0.91	0.01 (0; 0.02)	0.27	0.01 (0; 0.02)	0.45
City						
Manaus	Reference		Reference		Reference	
Rio de Janeiro	0.02 (-0.01; 0.05)	0.45	0.01 (-0.03; 0.04)	0.90	0 (-0.04; 0.04)	>0.99
Salvador	-0.01 (-0.04; 0.03)	0.85	-0.06 (-0.1; -0.02)	**0.044**	-0.01 (-0.06; 0.04)	0.85
Ethnicity						
Pardo	Reference		Reference		Reference	
Black	-0.01 (-0.04; 0.02)	0.86	0.01 (-0.03; 0.04)	0.89	0 (-0.05; 0.05)	>0.99
White	-0.03 (-0.06; 0)	0.37	0 (-0.04; 0.03)	0.95	-0.06 (-0.11; -0.02)	0.051
Indian	0 (-0.12; 0.13)	0.98	-0.12 (-0.26; 0.02)	0.37	-0.13 (-0.33; 0.06)	0.48
Asian	-0.06 (-0.32; 0.2)	0.84	0.13 (-0.12; 0.38)	0.60	-0.24 (-0.63; 0.15)	0.53
Smoking history						
Never smoked	Reference		Reference		Reference	
Former smoker	-0.01 (-0.04; 0.02)	0.80	-0.01 (-0.05; 0.03)	0.87	-0.02 (-0.07; 0.04)	0.82
Current smoker	0.03 (-0.01; 0.08)	0.39	0.06 (0.01; 0.11)	0.13	0.06 (-0.01; 0.12)	0.33
Drinking history						
Never drunk	Reference		Reference		Reference	
Former drinker	0.04 (0; 0.07)	0.23	0.03 (-0.01; 0.07)	0.47	-0.01 (-0.07; 0.04)	0.86
Current drinker	-0.01 (-0.03; 0.02)	0.86	0.03 (-0.01; 0.06)	0.45	-0.02 (-0.07; 0.02)	0.60
Baseline BMI (per 10 kg/m^2)	0.01 (-0.01; 0.03)	0.57	0.03 (0.01; 0.05)	0.089	-0.03 (-0.06; 0)	0.20
Baseline HIV status						
HIV negative	Reference		Reference		Reference	
HIV+ VL undetectable [b]	0.03 (-0.09; 0.15)	0.85	0.02 (-0.14; 0.18)	0.93	0.01 (-0.18; 0.2)	0.96
HIV+ VL detectable [b]	0.14 (0.03; 0.26)	0.089	0.29 (0.14; 0.44)	**0.001**	0.24 (0.05; 0.43)	0.073
Previous TB						
No	Reference		Reference		Reference	
Yes	0.01 (-0.05; 0.07)	0.86	0.04 (-0.04; 0.11)	0.62	0.04 (-0.05; 0.13)	0.66
Isoniazid preventive therapy						
Completed > 168 days	Reference		Reference		Reference	
Completed < 168 days	0.01 (-0.05; 0.06)	0.94	-0.05 (-0.12; 0.02)	0.45	0.06 (-0.03; 0.14)	0.53
No IPT end date [c]	0.1 (-0.02; 0.22)	0.35	-0.01 (-0.14; 0.13)	0.98	0.05 (-0.14; 0.23)	0.84
No IPT recorded [d]	0 (-0.04; 0.04)	0.95	0 (-0.04; 0.05)	0.94	0.04 (-0.02; 0.11)	0.48
Month 6 IGRA status [e]						
Persistent negative	Reference		Reference		Reference	
Baseline positive	-0.01 (-0.05; 0.02)	0.74	0.02 (-0.02; 0.06)	0.66	0.01 (-0.04; 0.07)	0.84
Converter (< 1 IU/mL)	0.01 (-0.04; 0.06)	0.84	0.02 (-0.04; 0.08)	0.82	-0.04 (-0.12; 0.04)	0.62
Converter (≥ 1 IU/mL)	0.05 (-0.02; 0.11)	0.46	0.06 (-0.03; 0.15)	0.47	0.11 (0.01; 0.21)	0.18
**Summary Statistics**						
Adjusted R^2	0.03		0.07		0.01	
Missing variables	322		373		383	
Degrees of freedom	1080		1029		1019	

[a] Correction for false discovery was performed using the Benjamini and Hochberg method.[26]
[b] HIV plasma viral load (VL) < 50 copies/mL was considered undetectable, and ≥ 50 copies/mL was considered detectable.
[c] Started isoniazid preventive therapy (IPT) during study, but no treatment end date recorded during the study.
[d] No isoniazid preventive therapy (IPT) recorded during the study.
[e] Interferon-γ release assay (IGRA) was only repeated among study participants with baseline negative (< 0.35 IU/mL) IGRA results. Participants with baseline and month 6 negative IGRA results are termed "persistent negative". "Converters" are those participants whose IGRA are negative at baseline, but convert to positive (≥ 0.35 IU/mL) at the month 6 visit. We divided the converters into those that converted their IGRA scores by less than (Converter; < 1 IU/mL) or greater than 1 IU/mL (Converter; ≥ 1 IU/mL).

BMI, body-mass index. HIV+, HIV positive. VL, viral load. IPT, isoniazid preventive therapy. IGRA, interferon-γ release assay.

Table S5. Transcriptomic signatures on the panel that were reparameterised.

Signature	Reference	Original gene expression measurement	Model used	HIV-negative training and test set (combined) AUC (95% CI)	HIV-positive validation set AUC (95% CI)
**Da Costa3**	1	RT-qPCR	Random forest	0.98 (0.93−1)	0.96 (0.93−1)
**Duffy9**	3	Meta-analysis of microarray data	Multinomial random forest	TB vs LTBI: 1 (1−1)*	
**Francisco2**	4	RT-qPCR	Random forest	0.99 (0.98−1)	0.83 (0.74−0.93)
**Gjøen7**	5	Dual-color-Reverse-Transcriptase-Multiplex-Ligation-dependent-Probe-Amplification (dc-RT MLPA)	LASSO regression	0.98 (0.94−1)	0.78 (0.67−0.88)
**Jacobsen3**	7	RT-qPCR	Linear discriminant analysis	0.99 (0.99−1)	0.95 (0.89−1)
**Roe3**	13	RNA sequencing	Support vector machines (linear kernel)	0.96 (0.89−1)	0.89 (0.80−0.97)
**Sambarey10**	14	RNA sequencing	Linear discriminant analysis	0.99 (0.96−1)	0.95 (0.90−1)
**Satproedprai7**	15	RT-qPCR	LASSO regression	0.99 (0.98−1)	0.95 (0.90 −1)

* HIV-positive samples required to parameterise the multinomial model. No independent validation set available.

Supplementary Figures

A

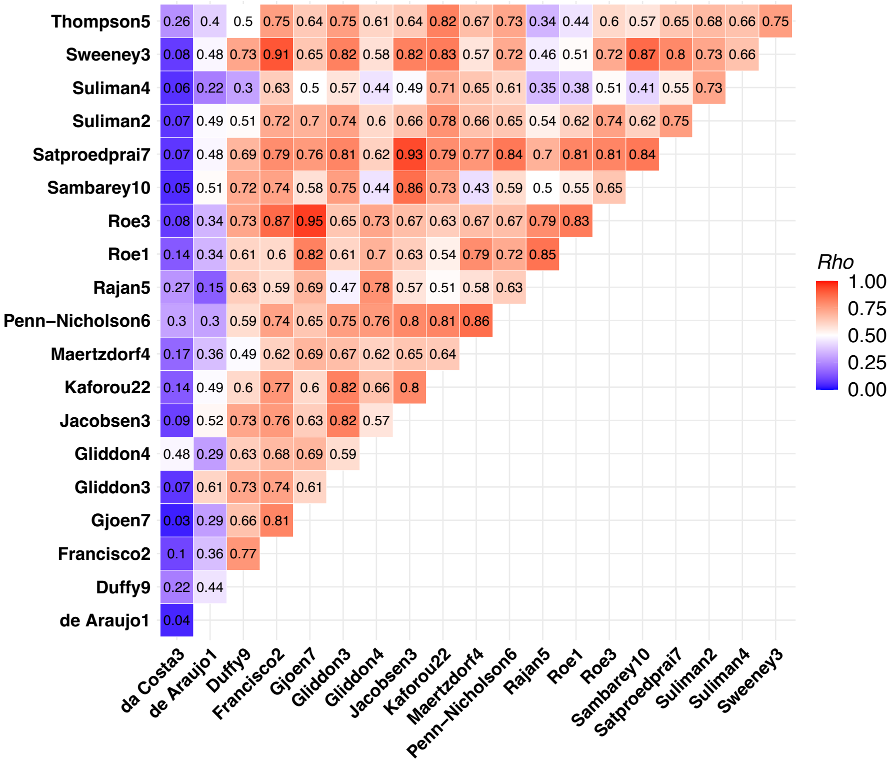

B

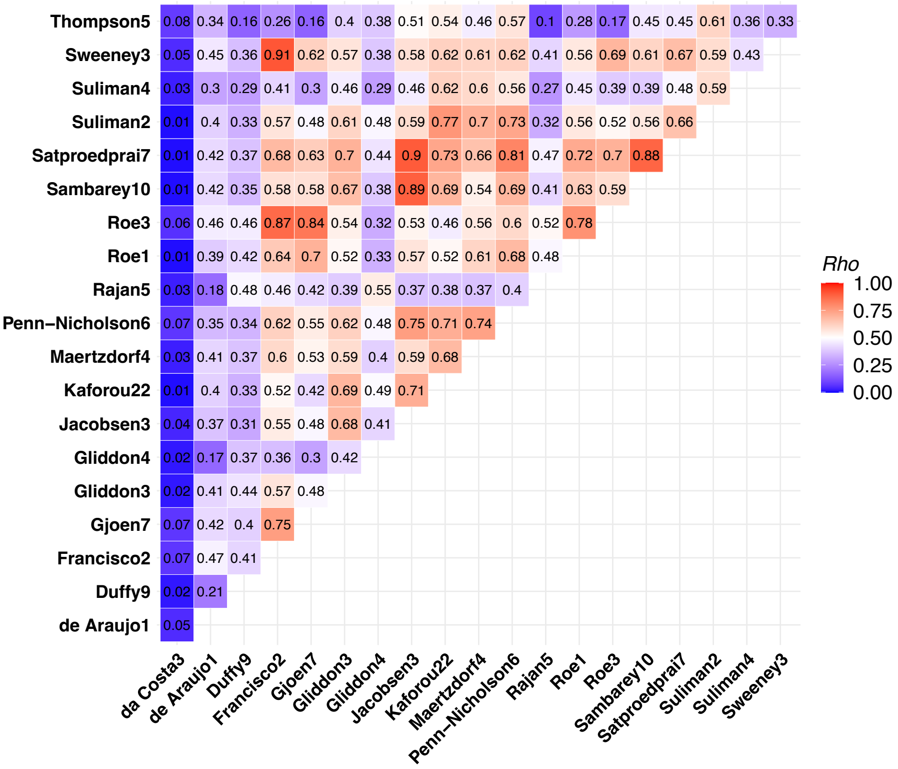

Figure S1. Correlation between signature scores.

Signature score correlation matrix with the Spearman-rank-order correlation coefficients (*rho*) among (**A**) progressors (n=26/39[#]) and (**B**) non-progressors (n=1,991/3,232[#]) with Month 0 and Month 6 sampling timepoints combined. De Araujo1, Rajan5, and Roe1 signatures were multiplied by −1 to obtain a positive correlation for all signatures. [#]Only participants with signature scores available for all signatures were included in the Spearman correlation analysis.

A

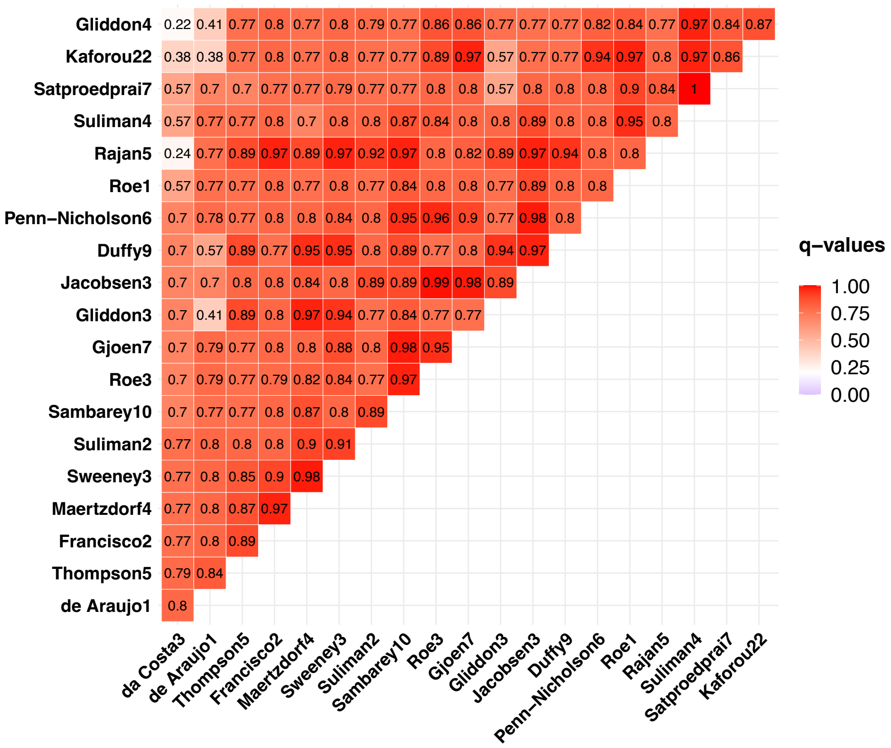
Gliddon4
Kaforou22
Satproedprai7
Suliman4
Rajan5
Roe1
Penn–Nicholson6
Duffy9
Jacobsen3
Gliddon3
Gjoen7
Roe3
Sambarey10
Suliman2
Sweeney3
Maertzdorf4
Francisco2
Thompson5
de Araujo1
da Costa3
de Araujo1
Thompson5
Francisco2
Maertzdorf4
Sweeney3
Suliman2
Sambarey10
Roe3
Gjoen7
Gliddon3
Jacobsen3
Duffy9
Penn–Nicholson6
Roe1
Rajan5
Suliman4
Satproedprai7
Kaforou22
q–values
1.00
0.75
0.50
0.25
0.00

B

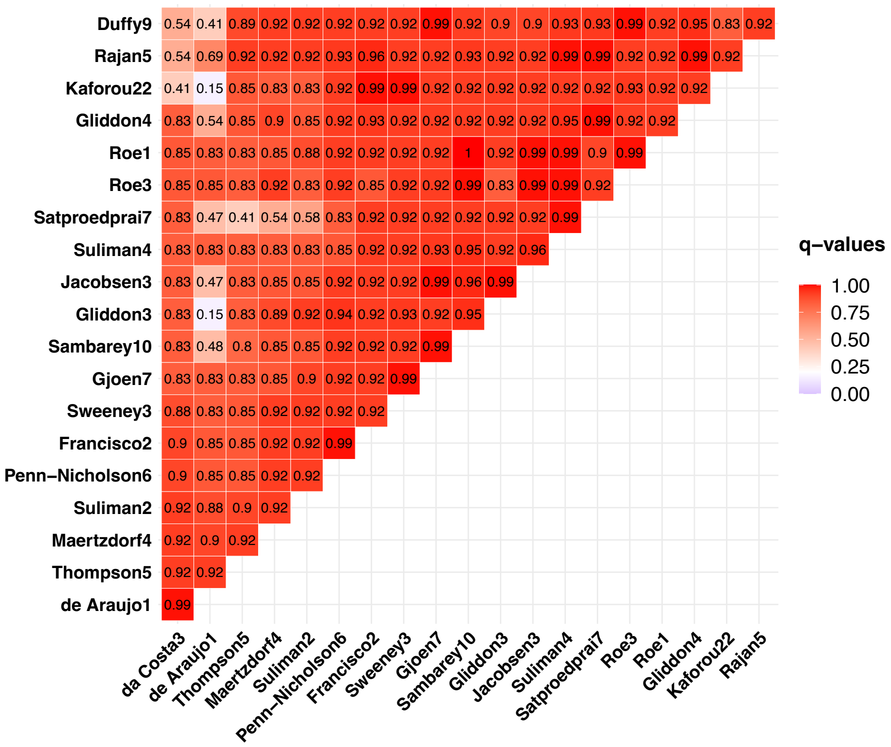
Duffy9
Rajan5
Kaforou22
Gliddon4
Roe1
Roe3
Satproedprai7
Suliman4
Jacobsen3
Gliddon3
Sambarey10
Gjoen7
Sweeney3
Francisco2
Penn–Nicholson6
Suliman2
Maertzdorf4
Thompson5
de Araujo1
da Costa3
de Araujo1
Thompson5
Maertzdorf4
Suliman2
Penn–Nicholson6
Francisco2
Sweeney3
Gjoen7
Sambarey10
Gliddon3
Jacobsen3
Suliman4
Satproedprai7
Roe3
Roe1
Gliddon4
Kaforou22
Rajan5
q–values
1.00
0.75
0.50
0.25
0.00

C

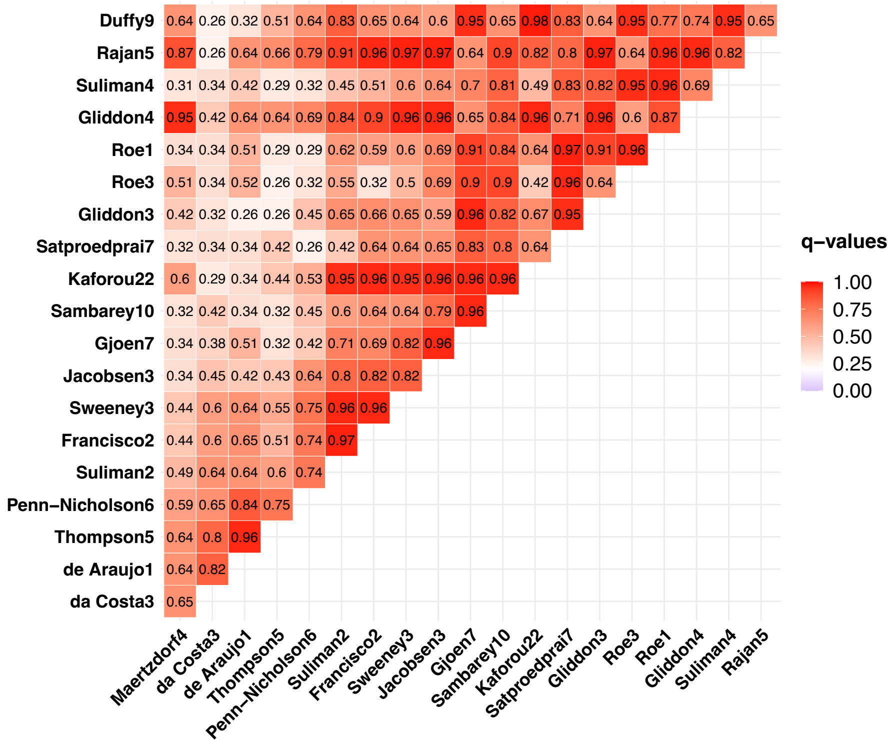

D

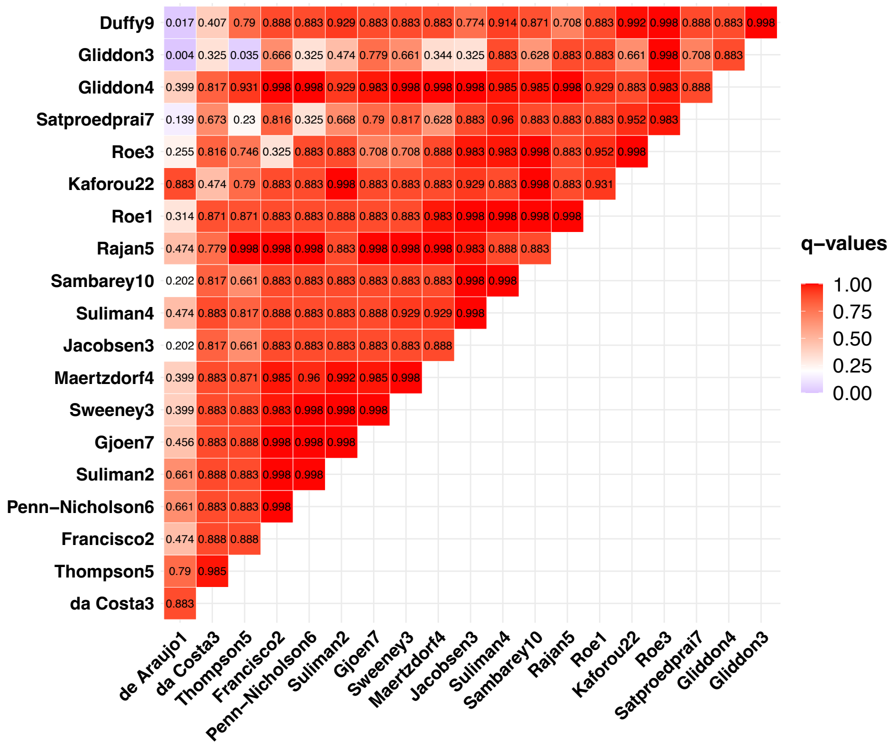

Figure S2. Comparison of transcriptomic signature prognostic performance through (A) 6, (B) 9, (C), 12, and (D) 24 months of follow-up.

Numbers in each square depict adjusted p-values (q-values), generated with the De Long method for comparing receiver operating characteristic curves computed for each pair of transcriptomic signatures, corrected for multiple comparisons using the Benjamini-Hochberg method.[26] Comparisons with adjusted p-values below 0.05 are shown in blue shading. Signatures on the y-axis and x-axis are listed in order of prognostic performance.

A

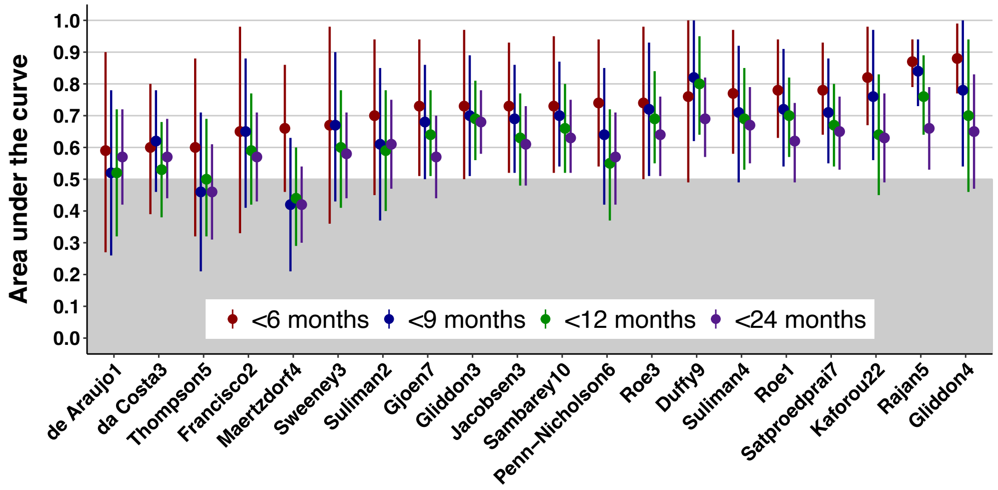
Area under the curve
1.0
0.9
0.8
0.7
0.6
0.5
0.4
0.3
0.2
0.1
0.0
<6 months
<9 months
<12 months
<24 months
de Araujo1
da Costa3
Thompson5
Francisco2
Maertzdorf4
Sweeney3
Suliman2
Gjoen7
Gliddon3
Jacobsen3
Sambarey10
Penn–Nicholson6
Roe3
Duffy9
Suliman4
Roe1
Satproedprai7
Kaforou22
Rajan5
Gliddon4

B

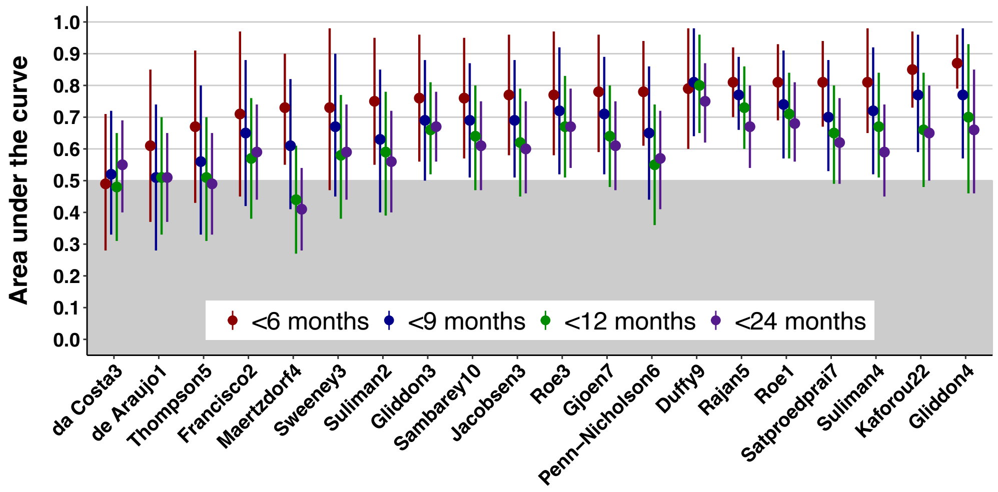
Area under the curve
1.0
0.9
0.8
0.7
0.6
0.5
0.4
0.3
0.2
0.1
0.0
<6 months
<9 months
<12 months
<24 months
da Costa3
de Araujo1
Thompson5
Francisco2
Maertzdorf4
Sweeney3
Suliman2
Gliddon3
Sambarey10
Jacobsen3
Roe3
Gjoen7
Penn–Nicholson6
Duffy9
Rajan5
Roe1
Satproedprai7
Suliman4
Kaforou22
Gliddon4

C

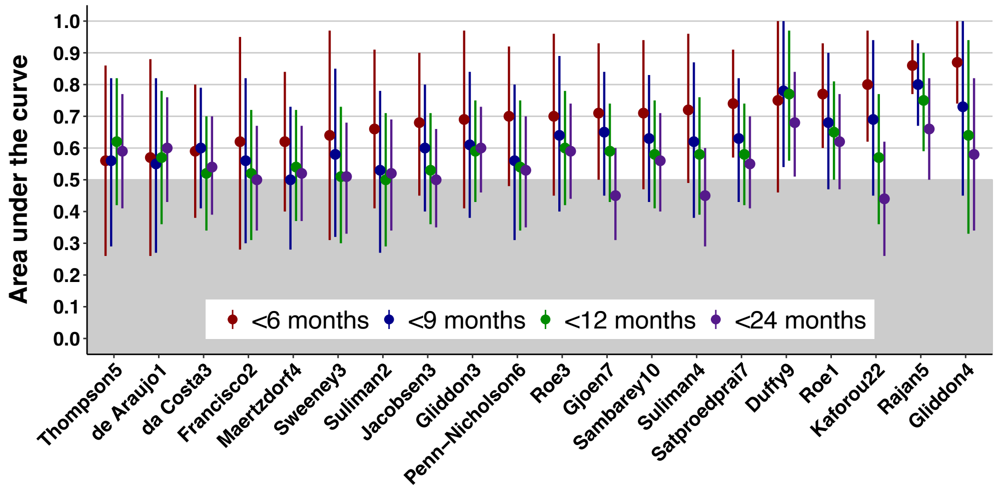
Area under the curve
1.0
0.9
0.8
0.7
0.6
0.5
0.4
0.3
0.2
0.1
0.0
<6 months
<9 months
<12 months
<24 months
Thompson5
de Araujo1
da Costa3
Francisco2
Maertzdorf4
Sweeney3
Suliman2
Jacobsen3
Gliddon3
Penn–Nicholson6
Roe3
Gjoen7
Sambarey10
Suliman4
Satproedprai7
Duffy9
Roe1
Kaforou22
Rajan5
Gliddon4

D

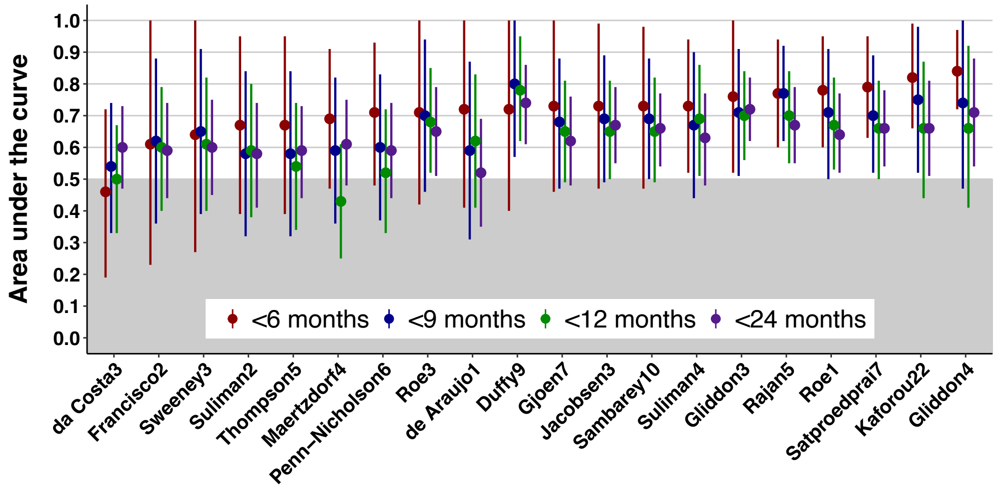

E

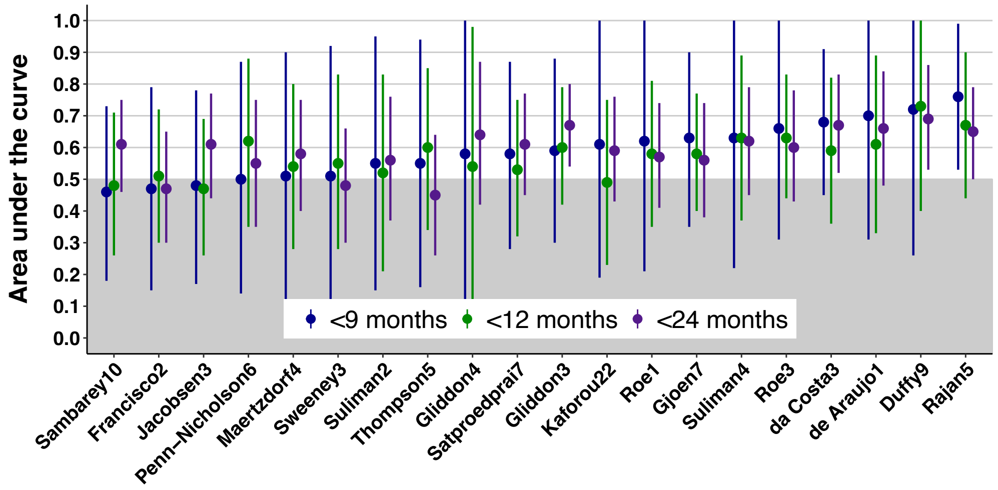

Figure S3. Sensitivity analyses of transcriptomic signature prognostic performance (**A**) among participants who did not receive IPT, (**B**) among IGRA positive participants, (**C**) among IGRA positive participants who did not receive IPT, (**D**) excluding clinically-diagnosed incident TB cases, and (**E**) excluding extra-pulmonary incident TB cases. Summary of signature prognostic performance in the order of receiver operating characteristic area under the curve (AUC) estimates through 6 months of follow up. The prognostic AUC estimates through 9, 12, and 24 months are also shown. The midline indicates the AUC estimate and the error bars indicate the 95% CIs.

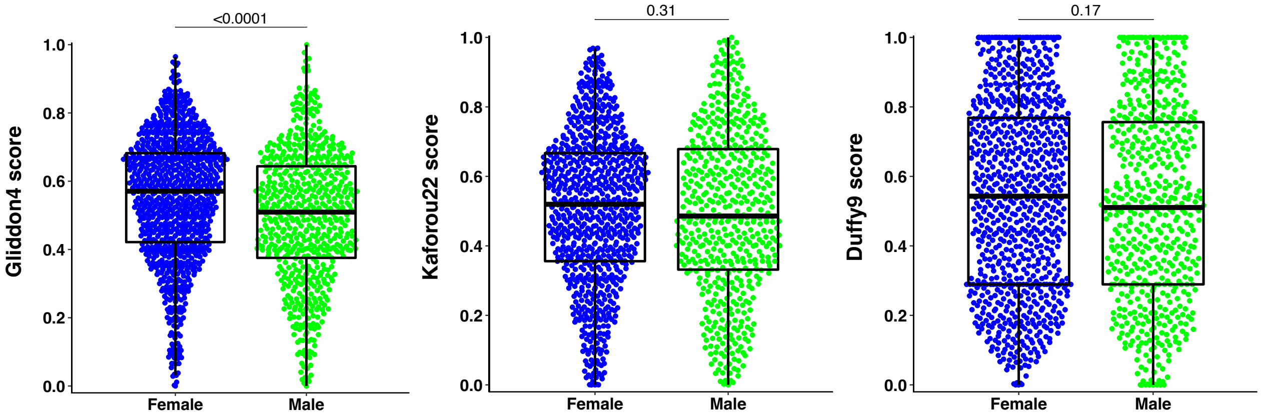

Figure S4. Gliddon4, Kaforou22, and Duffy9 signature scores at enrolment stratified by sex.

The box-and-whisker plots depict signature score (measured at enrolment) distribution among males and females (each dot represents a participant). p-values for comparison of median signature scores between groups in the box-and-whisker plot were calculated with the Mann–Whitney U test. Boxes depict the IQR, the midline represents the median, and the whiskers indicate the IQR ± (1.5 × IQR).

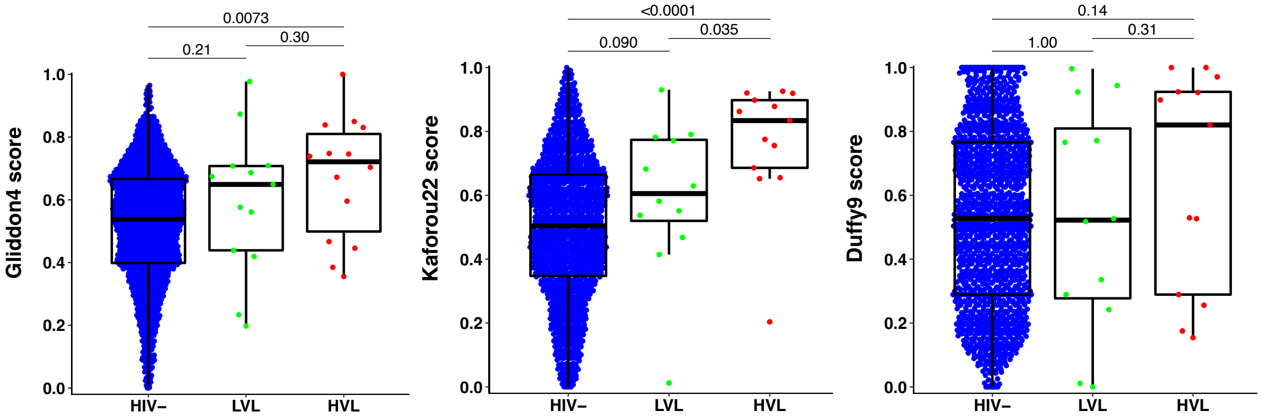

Figure S5. Gliddon4, Kaforou22, and Duffy9 signature scores at enrolment stratified by HIV status and HIV viral load.

The box-and-whisker plots depict signature score (measured at enrolment) distribution among people without HIV (HIV–) and those living with HIV with detectable and undetectable HIV viral load (each dot represents a participant). HIV plasma viral load < 50 copies/mL was considered undetectable (LVL, low viral load), and ≥ 50 copies/mL was considered detectable (HVL, high viral load). p-values for comparison of median signature scores between groups in the box-and-whisker plot were calculated with the Mann–Whitney U test. Boxes depict the IQR, the midline represents the median, and the whiskers indicate the IQR ± (1.5 × IQR).

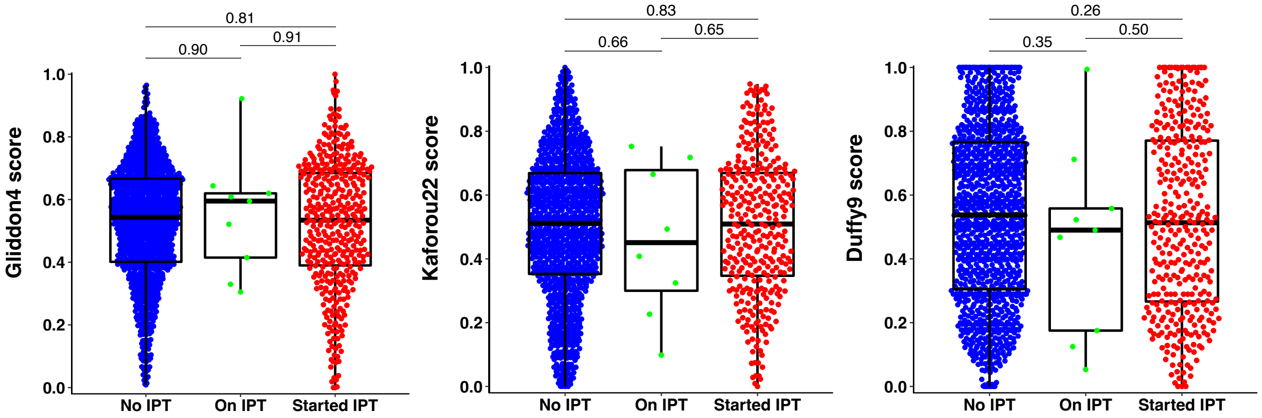

Figure S6. Gliddon4, Kaforou22, and Duffy9 signature scores stratified by IPT status at enrolment.

The box-and-whisker plots depict signature score (measured at enrolment) distribution (each dot represents a participant) among participants who were not on isoniazid preventive therapy (IPT) at enrolment, those already on IPT at enrolment, and those that started IPT during the course of the study. p-values for comparison of median signature scores between groups in the box-and-whisker plot were calculated with the Mann–Whitney U test. Boxes depict the IQR, the midline represents the median, and the whiskers indicate the IQR ± (1.5 × IQR).

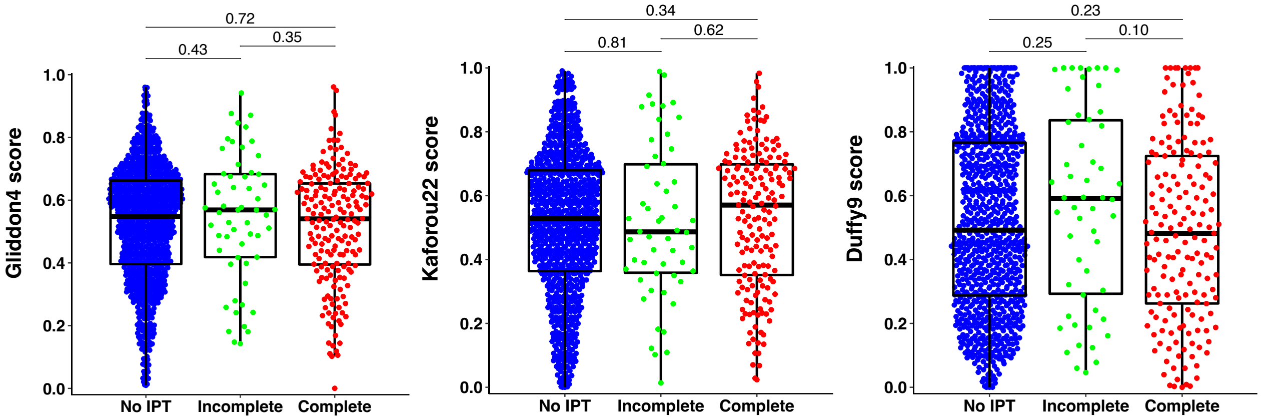

Figure S7. Gliddon4, Kaforou22, and Duffy9 signature scores stratified by IPT status at month 6 follow-up.

The box-and-whisker plots depict signature score (measured at 6 months of follow-up) distribution (each dot represents a participant) among participants who did not receive isoniazid preventive therapy (IPT) during the study, those that started but did not complete IPT during the study (Incomplete), and those that completed at least 6 months of IPT during the course of the study (Complete). p-values for comparison of median signature scores between groups in the box-and-whisker plot were calculated with the Mann–Whitney U test. Boxes depict the IQR, the midline represents the median, and the whiskers indicate the IQR ± (1.5 × IQR).

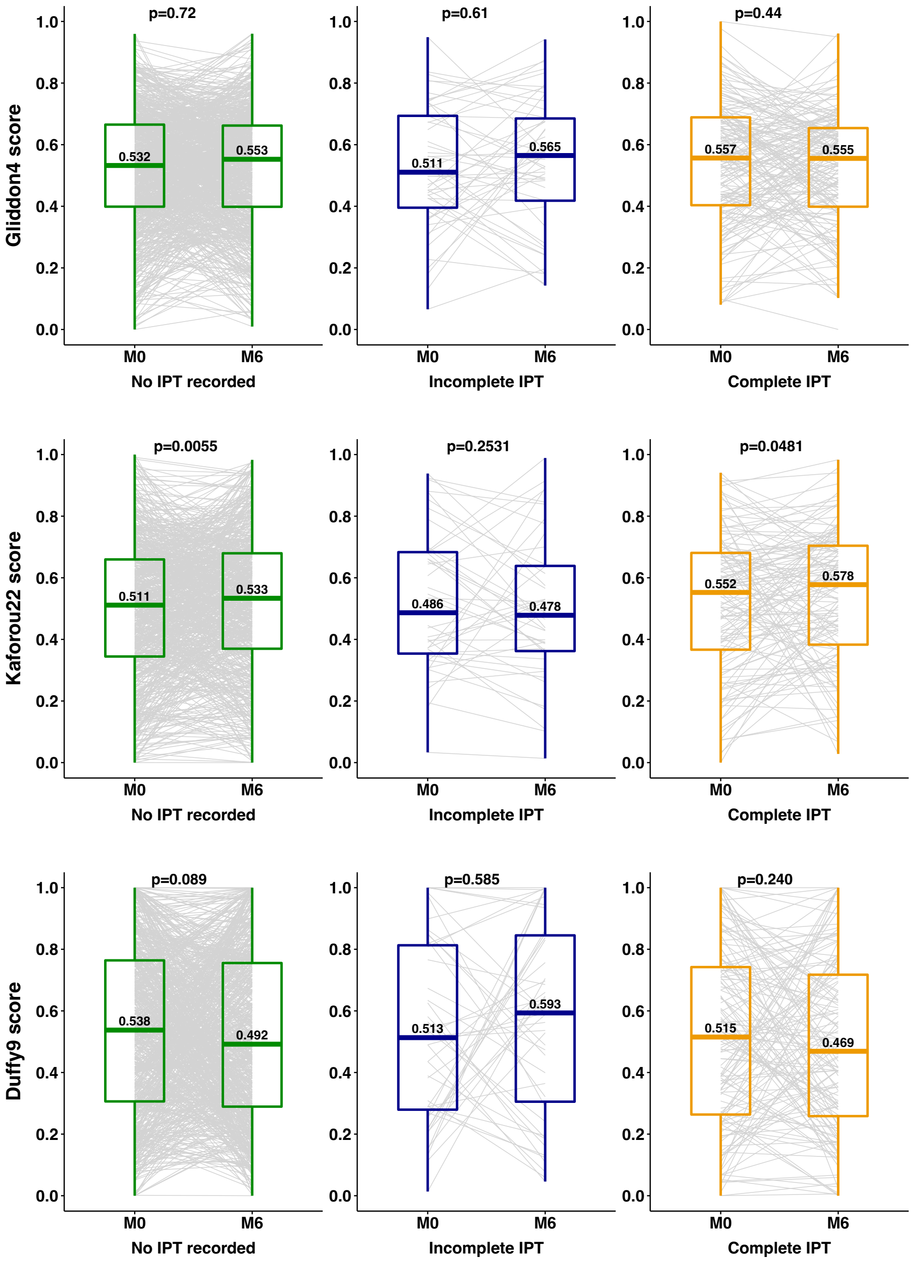

Figure S8. Change in Gliddon4, Kaforou22, and Duffy9 signature scores from baseline (M0) to month 6 (M6), stratified by IPT completion status at month 6 follow-up.

The box-and-whisker plots depict paired signature score (measured at enrolment and 6 months of follow-up) distribution (each grey line represents a participant) among participants who did not receive isoniazid preventive therapy (IPT) during the study, those that started but did not complete IPT during the study (Incomplete IPT), and those that completed at least 6 months of IPT during the course of the study (Complete IPT). p-values for comparison of median signature scores between groups in the box-and-whisker plot were calculated with the Wilcoxon signed-rank test. Boxes depict the IQR, the midline represents the median, and the whiskers indicate the IQR ± (1.5 × IQR).

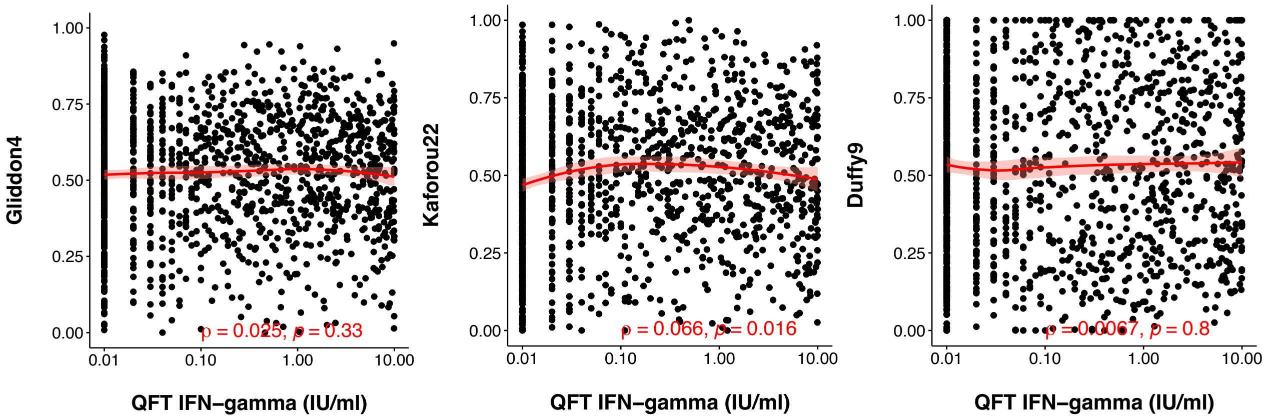

Figure S9. Correlation between IGRA IFN-γ response and Gliddon4, Kaforou22, and Duffy9 signature scores at enrolment.

Correlation between QuantiFERON TB-Gold interferon-γ (IFN-γ) response and signature scores measured at enrolment. The Spearman's rank correlation coefficient (rho, *p*) and p-value is shown. Each dot represents a participant, the locally estimated scatterplot smoothing (LOESS) curve represents the local polynomial regression, and the shaded area represents the 95% CI on the LOESS regression.

Supplementary References

1. da Costa LL, Delcroix M, Costa ERD, Prestes IV, Milano M, Francis SS, et al. A real-time PCR signature to discriminate between tuberculosis and other pulmonary diseases. Tuberculosis. 2015;95(4):421-5. doi: 10.1016/j.tube.2015.04.008.
2. de Araujo LS, Vaas LA, Ribeiro-Alves M, Geffers R, Mello FC, de Almeida AS, et al. Transcriptomic Biomarkers for Tuberculosis: Evaluation of DOCK9. EPHA4, and NPC2 mRNA Expression in Peripheral Blood. Front Microbiol. 2016;7:1586. doi: 10.3389/fmicb.2016.01586.
3. Duffy FJ, Thompson EG, Scriba TJ, Zak DE. Multinomial modelling of TB/HIV co-infection yields a robust predictive signature and generates hypotheses about the HIV+TB+ disease state. PLoS ONE. 2019;14(7):e0219322. doi: 10.1371/journal.pone.0219322.
4. Francisco NM, Fang YM, Ding L, Feng S, Yang Y, Wu M, et al. Diagnostic accuracy of a selected signature gene set that discriminates active pulmonary tuberculosis and other pulmonary diseases. Journal of Infection. 2017;75(6):499-510. doi: 10.1016/j.jinf.2017.09.012.
5. Gjoen JE, Jenum S, Sivakumaran D, Mukherjee A, Macaden R, Kabra SK, et al. Novel transcriptional signatures for sputum-independent diagnostics of tuberculosis in children. Sci Rep. 2017;7(1):5839. doi: 10.1038/s41598-017-05057-x.
6. Gliddon HD, Kaforou M, Alikian M, Habgood-Coote D, Zhou C, Oni T, et al. Identification of Reduced Host Transcriptomic Signatures for Tuberculosis Disease and Digital PCR-Based Validation and Quantification. Front Immunol. 2021;12:637164. doi: 10.3389/fimmu.2021.637164.
7. Jacobsen M, Repsilber D, Gutschmidt A, Neher A, Feldmann K, Mollenkopf HJ, et al. Candidate biomarkers for discrimination between infection and disease caused by Mycobacterium tuberculosis. J Mol Med (Berl). 2007;85(6):613-21. doi: 10.1007/s00109-007-0157-6.
8. Kaforou M, Wright VJ, Oni T, French N, Anderson ST, Bangani N, et al. Detection of tuberculosis in HIV-infected and -uninfected African adults using whole blood RNA expression signatures: a case-control study. PLoS Medicine. 2013;10(10):e1001538. doi: 10.1371/journal.pmed.1001538.
9. Maertzdorf J, McEwen G, Weiner J, 3rd, Tian S, Lader E, Schriek U, et al. Concise gene signature for point-of-care classification of tuberculosis. EMBO Molecular Medicine. 2016;8(2):86-95. doi: 10.15252/emmm.201505790.
10. Penn-Nicholson A, Mbandi SK, Thompson E, Mendelsohn SC, Suliman S, Chegou NN, et al. RISK6, a 6-gene transcriptomic signature of TB disease risk, diagnosis and treatment response. Sci Rep. 2020;10(1):8629. doi: 10.1038/s41598-020-65043-8.
11. Rajan JV, Semitala FC, Mehta T, Seielstad M, Montalvo L, Andama A, et al. A Novel, 5-Transcript, Whole-blood Gene-expression Signature for Tuberculosis Screening Among People Living With Human Immunodeficiency Virus. Clinical Infectious Diseases. 2019;69(1):77-83. doi: 10.1093/cid/ciy835.
12. Roe JK, Thomas N, Gil E, Best K, Tsaliki E, Morris-Jones S, et al. Blood transcriptomic diagnosis of pulmonary and extrapulmonary tuberculosis. JCI Insight. 2016;1(16):e87238. doi: 10.1172/jci.insight.87238.
13. Roe J, Venturini C, Gupta RK, Gurry C, Chain BM, Sun Y, et al. Blood Transcriptomic Stratification of Short-term Risk in Contacts of Tuberculosis. Clinical Infectious Diseases. 2020;70(5):731-7. doi: 10.1093/cid/ciz252.
14. Sambarey A, Devaprasad A, Mohan A, Ahmed A, Nayak S, Swaminathan S, et al. Unbiased Identification of Blood-based Biomarkers for Pulmonary Tuberculosis by Modeling and

Mining Molecular Interaction Networks. EBioMedicine. 2017;15:112-26. doi: 10.1016/j.ebiom.2016.12.009.
15. Satproedprai N, Wichukchinda N, Suphankong S, Inunchot W, Kuntima T, Kumpeerasart S, et al. Diagnostic value of blood gene expression signatures in active tuberculosis in Thais: a pilot study. Genes and Immunity. 2015;16(4):253-60. doi: 10.1038/gene.2015.4.
16. Suliman S, Thompson EG, Sutherland J, Weiner J, 3rd, Ota MOC, Shankar S, et al. Four-Gene Pan-African Blood Signature Predicts Progression to Tuberculosis. American Journal of Respiratory and Critical Care Medicine. 2018;197(9):1198-208. doi: 10.1164/rccm.201711-2340OC.
17. Sweeney TE, Braviak L, Tato CM, Khatri P. Genome-wide expression for diagnosis of pulmonary tuberculosis: a multicohort analysis. Lancet Respir Med. 2016;4(3):213-24. doi: 10.1016/S2213-2600(16)00048-5.
18. Warsinske HC, Rao AM, Moreira FMF, Santos PCP, Liu AB, Scott M, et al. Assessment of Validity of a Blood-Based 3-Gene Signature Score for Progression and Diagnosis of Tuberculosis, Disease Severity, and Treatment Response. JAMA Netw Open. 2018;1(6):e183779. doi: 10.1001/jamanetworkopen.2018.3779.
19. Thompson EG, Du Y, Malherbe ST, Shankar S, Braun J, Valvo J, et al. Host blood RNA signatures predict the outcome of tuberculosis treatment. Tuberculosis (Edinb). 2017;107:48-58. doi: 10.1016/j.tube.2017.08.004.
20. Muwanga V, Mendelsohn SC, Leukes V, Stanley K, Mbandi SK, Erasmus M, et al. Diagnostic performance of host blood transcriptomic signatures for pulmonary tuberculosis in a multi-cohort study of symptomatic patients in Africa. SSRN. 2023. doi: 10.2139/ssrn.4645194.
21. Scriba TJ, Fiore-Gartland A, Penn-Nicholson A, Mulenga H, Kimbung Mbandi S, Borate B, et al. Biomarker-guided tuberculosis preventive therapy (CORTIS): a randomised controlled trial. Lancet Infectious Diseases. 2021;21(3):354-65. doi: 10.1016/S1473-3099(20)30914-2.
22. Mendelsohn SC, Mbandi SK, Fiore-Gartland A, Penn-Nicholson A, Musvosvi M, Mulenga H, et al. Prospective multicentre head-to-head validation of host blood transcriptomic biomarkers for pulmonary tuberculosis by real-time PCR. Commun Med (Lond). 2022;2(1). doi: 10.1038/s43856-022-00086-8.
23. Darboe F, Mbandi SK, Naidoo K, Yende-Zuma N, Lewis L, Thompson EG, et al. Detection of Tuberculosis Recurrence, Diagnosis and Treatment Response by a Blood Transcriptomic Risk Signature in HIV-Infected Persons on Antiretroviral Therapy. Front Microbiol. 2019;10:1441. doi: 10.3389/fmicb.2019.01441.
24. Stopsack K, Gerke T. batchtma: Batch Effect Adjustments. 2021. https://CRAN.R-project.org/package=batchtma.
25. Signorell A, Aho K, Alfons A, Anderegg N, Aragon T, Arppe A, et al. DescTools: Tools for descriptive statistics. 2019. https://cran.r-project.org/web/packages/DescTools/index.html.
26. Benjamini Y, Hochberg Y. Controlling the False Discovery Rate - a Practical and Powerful Approach to Multiple Testing. Journal of the Royal Statistical Society Series B-Statistical Methodology. 1995;57(1):289-300. doi: DOI 10.1111/j.2517-6161.1995.tb02031.x.
27. Esmail H, Lai RP, Lesosky M, Wilkinson KA, Graham CM, Horswell S, et al. Complement pathway gene activation and rising circulating immune complexes characterize early disease in HIV-associated tuberculosis. Proceedings of the National Academy of Sciences of the United States of America. 2018;115(5):E964-E73. doi: 10.1073/pnas.1711853115.
28. Singhania A, Verma R, Graham CM, Lee J, Tran T, Richardson M, et al. A modular transcriptional signature identifies phenotypic heterogeneity of human tuberculosis infection. Nature Communications. 2018;9(1):2308. doi: 10.1038/s41467-018-04579-w.

29. Mendelsohn SC, Fiore-Gartland A, Penn-Nicholson A, Mulenga H, Mbandi SK, Borate B, et al. Validation of a host blood transcriptomic biomarker for pulmonary tuberculosis in people living with HIV: a prospective diagnostic and prognostic accuracy study. Lancet glob health. 2021;9(6):e841-e53. doi: 10.1016/S2214-109X(21)00045-0.
30. Mulenga H, Fiore-Gartland A, Mendelsohn SC, Penn-Nicholson A, Mbandi SK, Borate B, et al. The effect of host factors on discriminatory performance of a transcriptomic signature of tuberculosis risk. EBioMedicine. 2022;77:103886. doi: 10.1016/j.ebiom.2022.103886.
31. Wada PY, Costa AG, Araujo-Pereira M, Barreto-Duarte B, Souza AB, Rocha MS, et al. Possible sex difference in latent tuberculosis infection risk among close tuberculosis contacts. International Journal of Infectious Diseases. 2022;122:685-92. doi: 10.1016/j.ijid.2022.07.031.
